# The function of *OsbHLH068* is partially redundant with its homolog, *AtbHLH112*, in the regulation of the salt stress response but has opposite functions to control flowering in *Arabidopsis*

**DOI:** 10.1007/s11103-017-0624-6

**Published:** 2017-06-19

**Authors:** Hung-Chi Chen, Vicki Hsieh-Feng, Pei-Chun Liao, Wan-Hsing Cheng, Li-Yu Liu, Yun-Wei Yang, Ming-Hsin Lai, Men-Chi Chang

**Affiliations:** 1 Department of Agronomy, National Taiwan University, Taipei, Taiwan, ROC; 2 Institute of Plant and Microbial Biology, Academia Sinica, Taipei, Taiwan, ROC; 3 Crop Science Division, Taiwan Agricultural Research Institute, Taichung, Taiwan, ROC

**Keywords:** Transcription factor, OsbHLH068, AtbHLH112, Salt stress, Flowering

## Abstract

**Key message:**

The homologous genes OsbHLH068 and AtbHLH112 have partially redundant functions in the regulation of the salt stress response but opposite functions to control flowering in* Arabidopsis*.

**Abstract:**

The transcription factor (TF) basic/Helix-Loop-Helix (bHLH) is important for plant growth, development, and stress responses. *OsbHLH068*, which is a homologous gene of *AtbHLH112* that is up-regulated under drought and salt stresses, as indicated by previous microarray data analysis. However, the intrinsic function of *OsbHLH068* remains unknown. In the present study, we characterized the function and compared the role of *OsbHLH068* with that of its homolog, *AtbHLH112*. Histochemical GUS staining indicated that *OsbHLH068* and *AtbHLH112* share a similar expression pattern in transgenic *Arabidopsis* during the juvenile-to-adult phase transition. Heterologous overexpression of *OsbHLH068* in *Arabidopsis* delays seed germination, decreases salt-induced H_2_O_2_ accumulation, and promotes root elongation, whereas *AtbHLH112* knock-out mutant displays an opposite phenotype. Both *OsbHLH068*-overexpressing transgenic *Arabidopsis* seedlings and the *Atbhlh112* mutant display a late-flowering phenotype. Moreover, the expression of *OsbHLH068-GFP* driven by an *AtbHLH112* promoter can compensate for the germination deficiency in the *Atbhlh112* mutant, but the delayed-flowering phenotype tends to be more severe. Further analysis by microarray and qPCR indicated that the expression of *FT* is down-regulated in both *OsbHLH068*-overexpressing *Arabidopsis* plants and *Atbhlh112* mutant plants, whereas *SOC1* but not *FT* is highly expressed in *AtbHLH112*-overexpressing *Arabidopsis* plants. A comparative transcriptomic analysis also showed that several stress-responsive genes, such as *AtERF15* and *AtPUB23*, were affected in both *OsbHLH068*- and *AtbHLH112*-overexpressing transgenic *Arabidopsis* plants. Thus, we propose that OsbHLH068 and AtbHLH112 share partially redundant functions in the regulation of abiotic stress responses but have opposite functions to control flowering in *Arabidopsis*, presumably due to the evolutionary functional divergence of homolog-encoded proteins.

**Electronic supplementary material:**

The online version of this article (doi:10.1007/s11103-017-0624-6) contains supplementary material, which is available to authorized users.

## Introduction

The transcription factor (TF) basic/Helix-Loop-Helix (bHLH) protein comprises a large family in plants (Feller et al. 2011). To date, at least 162 *AtbHLHs* and 167 *OsbHLHs* have been identified and can be categorized into 25 subfamilies based on their bHLH domain (Bailey et al. 2003; Heim et al. 2003; Li et al. 2006; Toledo-Ortiz et al. 2003). A typical bHLH domain normally contains two functionally distinct regions: a basic region for DNA binding that recognizes the target *cis*-acting element, known as E-box (5′-CANNTG-3′), and an HLH region for protein homo- or hetero-dimerization (Feller et al. 2011; Heim et al. 2003; Murre et al. 1989; Pires and Dolan 2010). Notably, a few atypical members known as HLHs lack the basic region. Thus, the HLH–bHLH heterodimeric complex can disrupt the bHLH–bHLH interaction and prevent DNA binding. Based on the dimeric forms, plant bHLH can play a dual role in regulating growth and development. For example, the POSITIVE REGULATOR OF GRAIN LENGTH 1 (PGL1)-ANTAGONIST OF PGL1 (APG) heterodimer, an OsHLH–OsbHLH complex, increases grain length and weight, whereas the APG homodimer decreases grain length and weight (Heang and Sassa 2012). Moreover, plant bHLHs are also involved in many aspects of growth and development, including Z-box binding factor 1 (ZBF1/AtbHLH6), dysfunctional tapetum 1 (DYT1/AtbHLH22), and root hairless 1 (RHL1/OsbHLH3), which are involved in blue light-mediated seedling development, anther development, and root hair development, respectively (Cui et al. 2016; Ding et al. 2009; Maurya et al. 2015). However, the physiological and regulatory roles of most bHLHs in either *Arabidopsis* or rice remain poorly understood.

Although plants are sessile organisms that cannot escape from deleterious environments, they have already developed and established an exquisite regulatory mechanism to address the changes in their surrounding environment. For example, the inducer of CBF expression 1 (ICE1)-*C-repeat binding factor 3*/*dehydration-responsive element-binding protein 1 A* (*CBF3*/*DREB1A*) transcriptional cascade, a well-known bHLH–*APETALA2*/*ethylene-responsive factor* (*AP2*/*ERF*) regulatory pathway, contributes to cold tolerance in *Arabidopsis* and rice, which illustrates the importance of bHLH in plant stress responses (Chinnusamy et al. 2003; Ito et al. 2006). In fact, AtbHLH116/ICE1 positively regulates the expression of *CBF3*/*DREB1A* [*AtERF#031* (At4g25480)], which confers freezing tolerance through an ABA-independent pathway (Chinnusamy et al. 2003). In addition to AtbHLH116/ICE1, several plant bHLHs are also involved in abiotic stress responses. For example, OrbHLH1 and OrbHLH2, two ICE-like proteins in wild rice (*Oryza rufipogon*), confer salt stress tolerance in transgenic *Arabidopsis* plants through an ICE/CBF-independent and an ABA-independent pathway, respectively (Li et al. 2010; Zhou et al. 2009). OsbHLH062 activates the expression of JA-responsive genes to confer salt stress tolerance, while OsbHLH062 represses the expression of JA-responsive genes when interacting with JASMONATE-ZIM-DOMAIN PROTEIN 9 (OsJAZ9) and NOVEL INTERACTOR OF JAZ (OsNINJA) (Wu et al. 2015). NaCl-induced expression of *At* *bHLH092* confers tolerance to salt and osmotic stresses in *Arabidopsis* through a pathway that is partially dependent on ABA and *SALT OVERLY SENSITIVE 2* (*SOS2*) (Jiang et al. 2009). In response to the high ambient temperature, AtbHLH9/PHYTOCHROME INTERACTING FACTOR 4 (PIF4) directly activates the expression of *YUCCA8* and *TAA1*, two auxin biosynthetic genes, which in turn triggers hypocotyl elongation in *Arabidopsis* (for a review, see Proveniers and van Zanten 2013). AtbHLH122 improves drought and osmotic tolerance through direct repression of *CYP707A3*, an ABA catabolic gene (Liu et al. 2014). Interestingly, AtbHLH112, which belongs to the F subfamily, confers abiotic stress tolerance by increasing proline levels and enhancing reactive oxygen species (ROS) scavenging ability; however, overexpression of *AtbHLH112* suppresses lateral root emergence (Liu et al. 2015; Wang et al. 2014). These studies reveal that some bHLHs, such as AtbHLH9/PIF4 and AtbHLH112, are not only involved in the stress response but also play a pleiotropic regulatory role for optimal plant growth and development .

Flowering time is one of the major yield traits correlated with grain production in cereal crops. The precise timing of flowering in plants is coordinately controlled by endogenous and environmental factors, including physiological maturity, accumulated temperature, and day length. In *Arabidopsis*, a facultative long-day (LD) plant, flowering time is determined by the autonomous, vernalization, photoperiod, and gibberellin pathways (Mouradov et al. 2002), during which photoperiod is the predominant cue controlling flowering time (Andrés and Coupland 2012; Song et al. 2015). These four major pathways integratedly regulate the expression of floral meristem identity genes, such as *FLOWERING LOCUS T* (*FT*), *SUPPRESSOR OF OVEREXPRESSION OF CONSTANS 1* (*SOC1*), and *LEAFY* (*LFY*), to trigger floral initiation (Wigge et al. 2005). Compared to *Arabidopsis*, rice is a facultative short-day (SD) plant and thus flowers earlier under SDs than LDs (Hayama et al. 2003). In rice, two *FT* homologous genes, *Heading date 3a* (*Hd3a*) and *RICE FLOWERING LOCUS T 1* (*RFT1*), encode a ‘florigen’ that promotes flowering (Komiya et al. 2008, 2009; Tamaki et al. 2007; Tsuji et al. 2013). The expression of *Hd3a* and *RFT1* is regulated by Early heading date 1 (Ehd1) and Heading date 1 (Hd1) (Doi et al. 2004). In fact, Ehd1 activates the expression of *Hd3a* and *RFT1* under both SDs and LDs, whereas Hd1 functions as a transcriptional activator of *Hd3a* under SDs but not under LDs (Hayama et al. 2003; Ishikawa et al. 2011). Recently, several plant bHLHs have also been found to be involved in the control of flowering. Four FLOWERING BHLH (FBH1-4) proteins directly activate *CONSTANS* (*CO*) expression for photoperiodic flowering in *Arabidopsi*s (Ito et al. 2012). Under SDs, NO FLOWERING IN SHORT DAY (NFL)/bHLH093 promotes flowering through the GA signaling pathway (Sharma et al. 2016). In rice, *Oryza sativa* late flowering (OsLF), an atypical HLH (OsbHLH119), directly represses *Hd1* expression to delay flowering (Wang et al. 2013; Zhao et al. 2011). However, to date, almost no typical OsbHLHs have been shown to regulate flowering.

Flowering time is strongly correlated with environmental factors in plants (for a review, see Riboni et al. 2014). Modification of flowering time for plants to set seeds under a stressful environment is an important survival mechanism for the continuation of the species. Previous studies have indicated that TFs play a vital role in connecting environmental factors to plant flowering. For example, *Oryza sativa* ABA responsive element binding factor 1 (OsABF1), a drought-inducible bZIP TF, directly activates the expression of *OsWRKY104*, which suppresses the expression of *Edh1* to delay rice flowering under water deficiency (Zhang et al. 2016). Although several OsbHLHs regulate growth, development, or stress responses, the pleiotropic effect of typical OsbHLH on both the stress response and flowering has not yet been reported. In the present study, our data reveal that the function of OsbHLH068 in the regulation of salt stress responses is partially redundant with its homolog, AtbHLH112, but acts oppositely to control flowering in *Arabidopsis*, presumably due to a functional divergence of the homolog-encoded proteins during evolution.

## Results

### Salt enhances OsbHLH068 expression

**Fig. 1:**
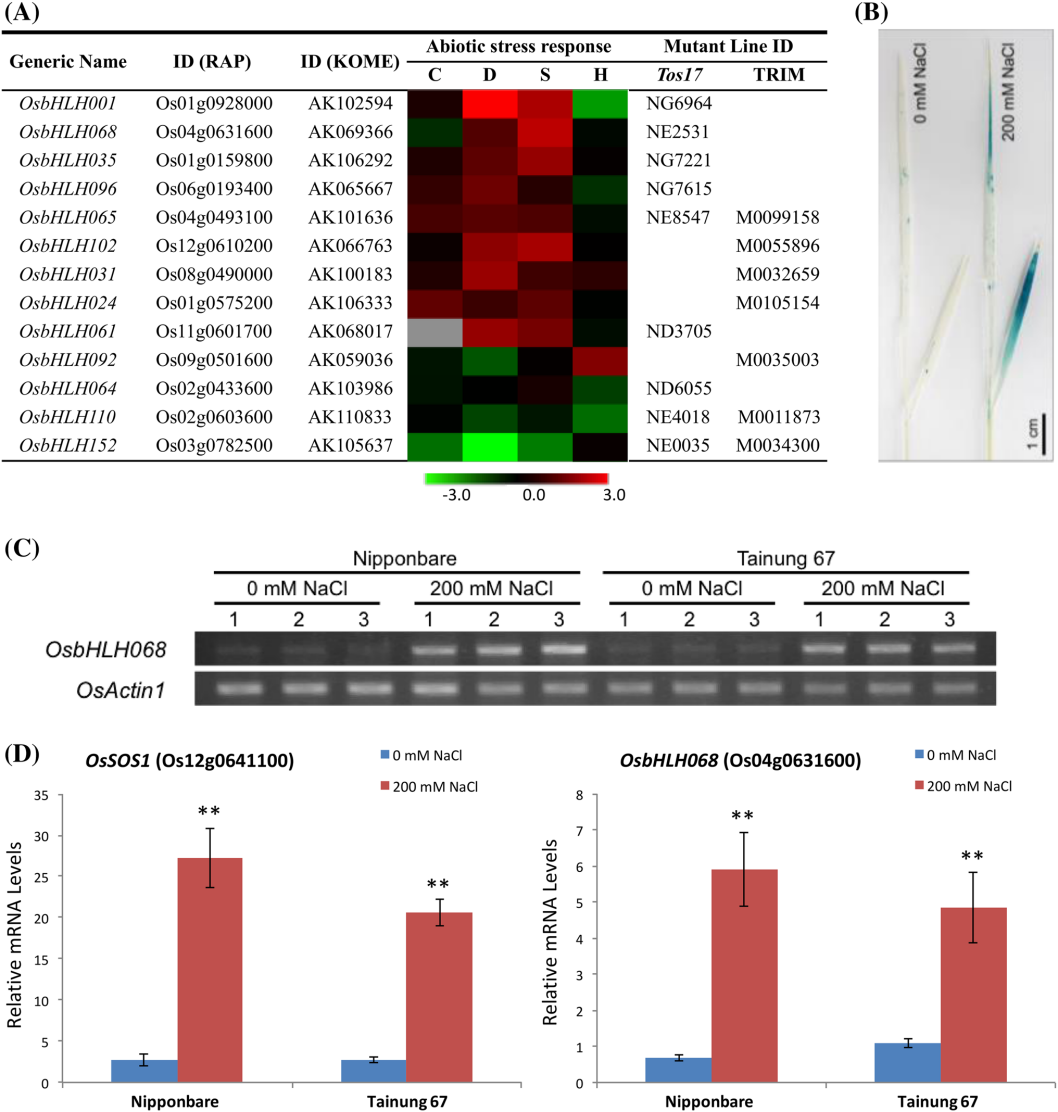
Salt-enhanced *OsbHLH068* expression. **a** Abiotic stress-responsive *OsbHLHs. Red and green* indicate increased and decreased gene expression, respectively. The scale bar shows log_2_-fold changes. C, cold treatment; D, drought treatment; H, heat treatment; S, salt treatment. *Tos17*, rice *Tos17* insertion mutant database; TRIM, Taiwan rice insert mutant database. **b** Histochemical staining of the aerial tissues in *OsbHLH068p::GUS* transgenic Tainung 67 (*Oryza sativa* L. *spp. japonica* cv. Tainung 67) seedlings. **c** The expression pattern of *OsbHLH068* in Nipponbare (*Oryza sativa* L. spp. *japonica* cv. Nipponbare) and Tainung 67 aerial tissues by RT-PCR. The Arabic numerals represent the individual rice plants. **d** Quantification of *OsSOS1* and *OsbHLH068* mRNA levels in Nipponbare and Tainung 67 aerial tissues by qPCR. The values are the mean ± SD of two independent experiments, each performed in triplicate. *P < 0.05; **P < 0.01, Student’s *t*-test. Seedlings used for GUS staining (**b**), RT-PCR (**c**), and qPCR (**d**) assays were grown on basal medium for 13 days and then transferred to basal medium containing 0 or 200 mM NaCl for an additional day

To determine which uncharacterized *OsbHLH* members may also be involved in regulating abiotic stress responses, we analyzed publicly available microarray data (GSE6901), and several abiotic stress-responsive *OsbHLH*s with an unknown function were identified (Fig. 1a). Among them, the expression of *OsbHLH068* was up-regulated under either drought- or salt-treated conditions. Notably, OsbHLH068 and AtbHLH112 have been categorized as members of the F subfamily (Li et al. 2006). Phylogenetic analysis also revealed that OsbHLH068 was highly homologous to AtbHLH112 based on the sequence similarity within the bHLH domains (Supplementary Fig. S1).

**Fig. 2:**
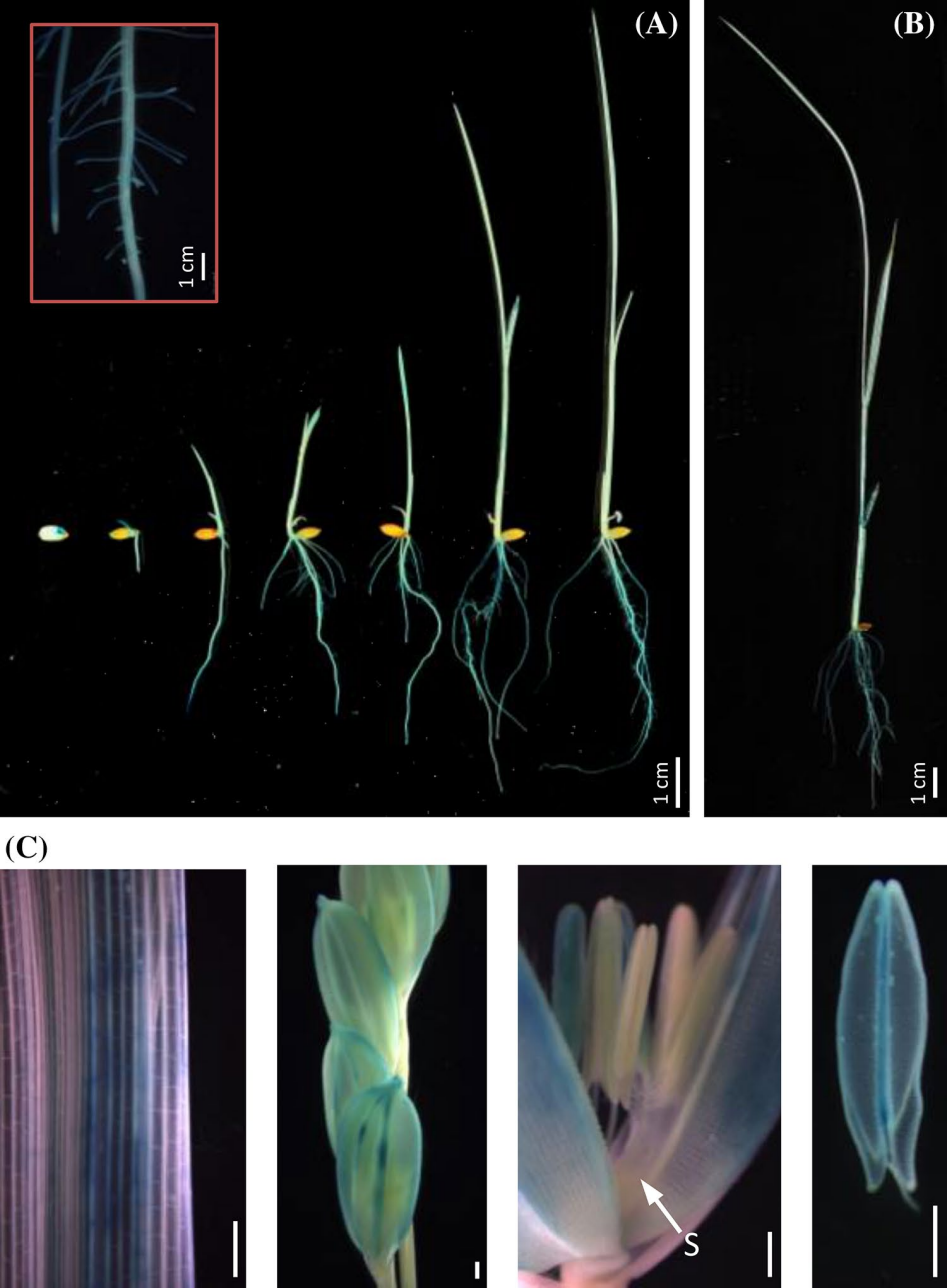
Spatiotemporal expression of the *OsbHLH068* gene. **a** Post-germination to second-leaf stages; **b** third-leaf stage; **c** reproductive stage. *Scale bar* 0.5 cm; S stigma

To verify whether the expression of *OsbHLH068* was in fact up-regulated under salt stress conditions, histochemical staining using *OsbHLH068p::GUS* transgenic rice was performed. Under 0 mM NaCl conditions, *GUS* was slightly expressed in the aerial tissues of 14-day-old transgenic seedlings; however, it was enhanced in response to 200 mM NaCl (Fig. 1b). Additionally, the expression pattern of *OsbHLH068* was similar to that of *OsSOS1*, a well-known salt-responsive gene that was up-regulated under 200 mM NaCl conditions in Nipponbare and Tainung 67 seedlings (Fig. 1c, d). These data show that *OsbHLH068*, an *AtbHLH112* homolog, is a salt-responsive gene. Incidentally, we also investigated the spatiotemporal expression of *OsbHLH068* in rice. As shown in Fig. 2a, b, the GUS signals were localized to the embryo of germinated transgenic seeds and in the terrestrial tissues of transgenic seedlings. Additionally, GUS signals were also observed in flag leaves, lemma nerves, and anthers during reproductive growth (Fig. 2c).

### Overexpression of OsbHLH068 confers salt tolerance in Arabidopsis

**Fig. 3:**
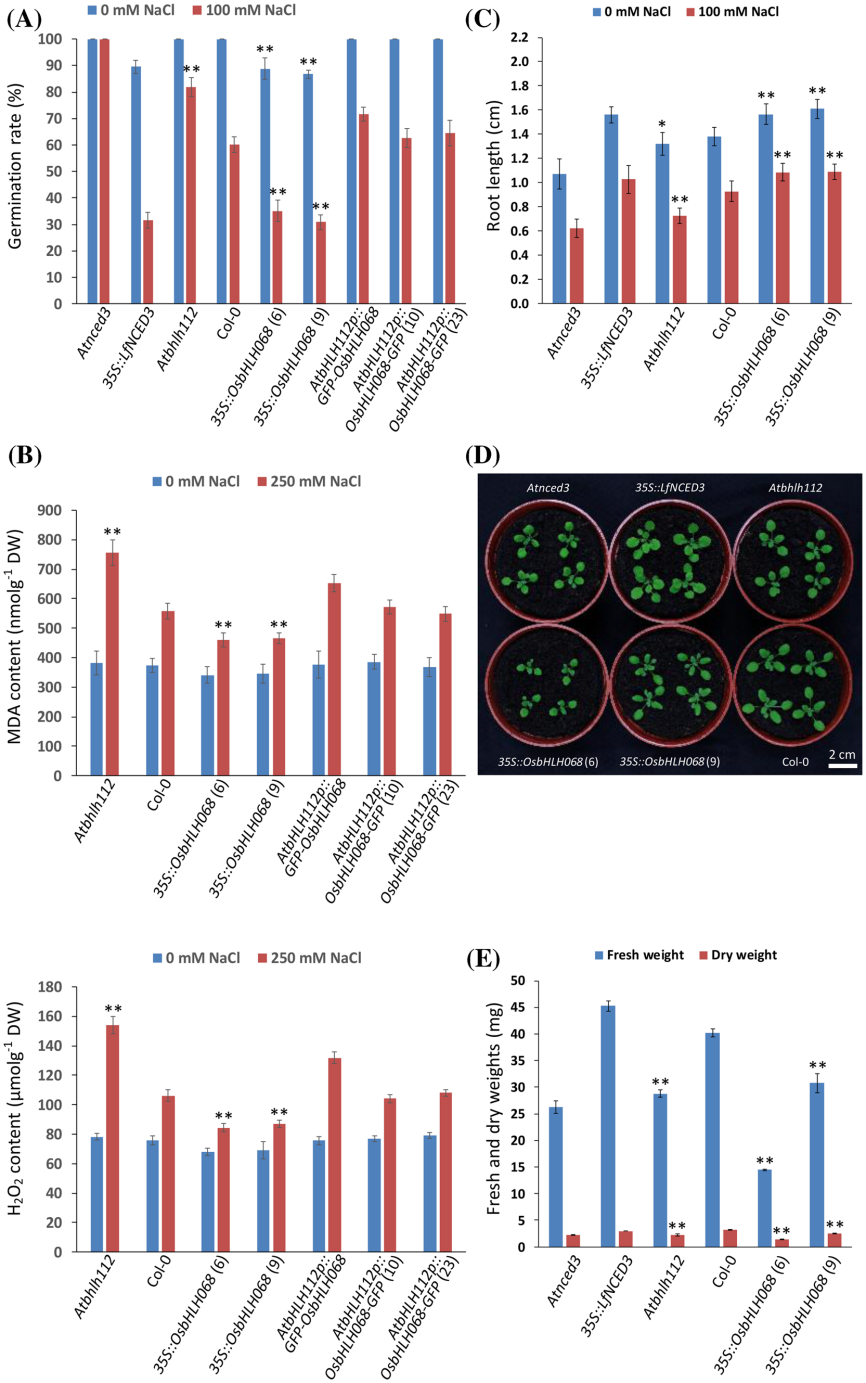
Seed germination, physiological investigation, root elongation, and plant morphology in heterologous *OsbHLH068*-overexpressing transgenic *Arabidopsis* and the complemented transformant. **a** The seed germination rates in the *Atnced3* mutant, *35S::LfNCED3* transformant, *Atbhlh112* mutant, Col-0, two *OsbHLH068*-overexpressing transgenic *Arabidopsis* lines, and three complemented transformants on day 2. **b** The levels of MDA (upper panel) and H_2_O_2_ (*lower panel*) in the *Atbhlh112* mutant, Col-0, two *OsbHLH068*-overexpressing transgenic *Arabidopsis* lines, and three complemented transformants. **c** The root lengths of the *Atnced3* mutant, *35S::LfNCED3* transformant, *Atbhlh112* mutant, Col-0, and two *OsbHLH068*-overexpressing transgenic *Arabidopsis* lines. **d** Phenotypic comparison of the 15-day-old *Atnced3* mutant, *35S::LfNCED3* transformant, *Atbhlh112* mutant, Col-0, and two *OsbHLH068*-overexpressing transgenic *Arabidopsis* lines grown under LD conditions. **e** The fresh and dry weights of *Atnced3* mutant, *35S::LfNCED3* transformant, *Atbhlh112* mutant, Col-0, and two *OsbHLH068*-overexpressing transgenic *Arabidopsis* lines. *P < 0.05; **P < 0.01, Student’s *t*-test. The ABA-deficient mutant, *Atnced3*, and the ABA over-accumulating *35S::LfNCED3* transformant were used as negative and positive controls, respectively. *Atbhlh112* mutant, SALK_148540

To investigate whether OsbHLH068 plays a positive regulatory role in the salt stress response similar to that of its homolog, AtbHLH112, we compared the seed germination properties of *Atbhlh112*, Col-0, two *OsbHLH068*-overexpressing transgenic *Arabidopsis* lines [*35S::OsbHLH068* (6) and (9)], and three independent complemented transformants [*AtbHLH112p::GFP-OsbHLH068, AtbHLH112p::OsbHLH068-GFP* (10), and (23)] sown on basal medium containing 0 or 100 mM NaCl. Genetic identification of homozygous *Atbhlh112* mutant lines (SALK_033618 and _148540), *OsbHLH068*-overexpressing transgenic *Arabidopsis* lines, and complemented transformants was performed by genomic DNA genotyping (Supplementary Fig. S2 and S3). Similar to the ABA over-accumulating *35S::LfNCED3* transformant, two independent *OsbHLH068*-overexpressing transgenic *Arabidopsis* lines had a lower germination rate in the presence of either 0 or 100 mM NaCl on day 2 compared with the corresponding Col-0 (Fig. 3a). The germination rate of the *Atbhlh112* mutant was not significantly different from that of Col-0 in the presence of 0 mM NaCl on day 2 but increased in the mutant compared with Col-0 under 100 mM NaCl conditions (82% and 60%, respectively). On day 3, almost all of the seeds had germinated under 0 mM NaCl conditions; however, two *OsbHLH068*-overexpressing transgenic lines and the *35S::LfNCED3* transformant still exhibited a lower germination rate under 100 mM NaCl conditions (Supplementary Fig. S4A). Notably, the germination rates of the three independent complemented transformants ranged from approximately 63–72% under 100 mM NaCl conditions on day 2; these rates were similar to the corresponding levels in Col-0 (Fig. 3a). These results indicate that OsbHLH068 can replace the functional role of AtbHLH112 in the regulation of seed germination. On 200 mM NaCl-containing medium, the *Atbhlh112* had a higher germination rate than the corresponding Col-0 at days 2 and 3 after seedling, whereas the germination rates of two *OsbHLH068*-overexpressing transgenic lines were lower than that of the corresponding Col-0 at days 2, 3, 4, and 5 after seedling (Supplementary Fig. S4B). Seed germination reached approximately 100% in each genotype at day 6, reflecting that seed germination delay in *OsbHLH068*-overexpressing transgenic lines was not due to poor seed vitality. Although *Atbhlh112* displayed early germination on 200 mM NaCl-containing medium, more bleached *Atbhlh112* seedlings were observed after prolonged culture; however, the late-germinating *OsbHLH068*-overexpressing transgenic lines had a lower bleached seedling frequency than both Col-0 and *Atbhlh112* (Supplementary Fig. S4C, D). Additionally, further investigations revealed that the two *OsbHLH068*-overexpressing transgenic lines had a lower level of both malondialdehyde (MDA, a lipid peroxidation marker) and hydrogen peroxide (H_2_O_2_, a ROS) than the corresponding Col-0 under salt (250 mM NaCl) treatment conditions, whereas the *Atbhlh112* mutant had a relatively high level of both MDA and H_2_O_2_ (Fig. 3b). Moreover, the levels of both MDA and H_2_O_2_ in the complemented transformants were similar to the corresponding levels in Col-0 under salt treatment conditions. Taken together, these data show that OsbHLH068 plays a similar role to that of AtbHLH112 in the regulation of the plant response to salt stress.

In addition to the salt stress response, previous studies have shown that overexpression of *AtbHLH112* suppresses lateral root emergence and salt-inhibited root growth (Liu et al. 2015; Wang et al. 2014). Therefore, we also investigated whether overexpression of *OsbHLH068* affects root development. Indeed, two independent *OsbHLH068*-overexpressing transgenic *Arabidopsis* lines and the *35S::LfNCED3* transformant had a longer primary root compared with the corresponding Col-0 seedlings under either 0 or 100 mM NaCl conditions on day 5, whereas the primary roots of both the *Atbhlh112* and ABA-deficient *Atnced3* mutants were shorter (Fig. 3c). Additionally, the fresh and dry weights of *OsbHLH068*-overexpressing transgenic *Arabidopsis* aerial tissues were less than those of Col-0 when grown in soil on day 15 (Fig. 3d, e).

### OsbHLH068 and AtbHLH112 act oppositely to control flowering in Arabidopsis

**Fig. 4:**
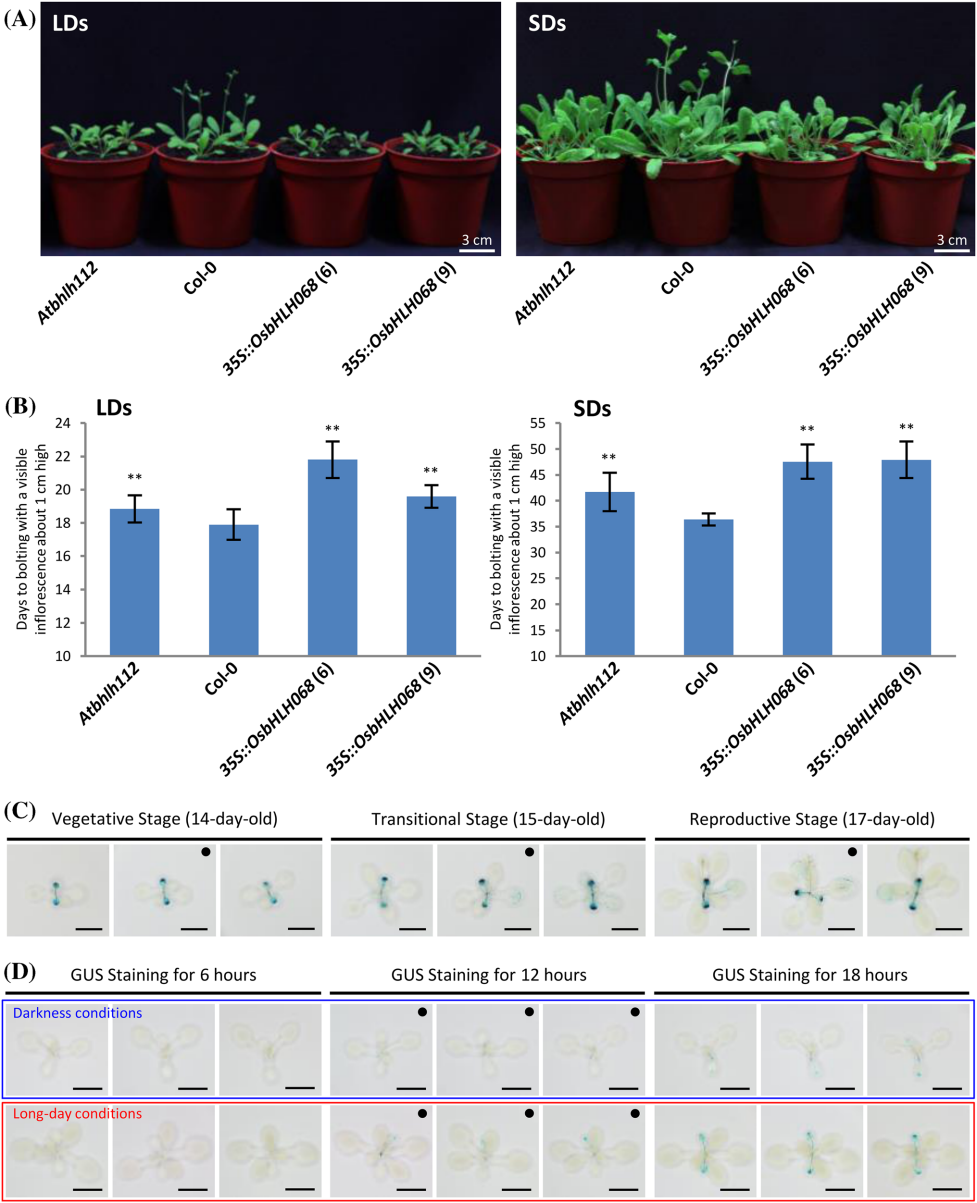
Heterologous *OsbHLH068*-overexpressing transgenic *Arabidopsis* plants display a late-flowering phenotype. **a** Phenotypic comparison of the *Atbhlh112* mutant, Col-0, and two *35S::OsbHLH068* transformants grown under LD (*left*) and SD (*right*) conditions. Seedlings were grown under LD and SD conditions for 19 and 41 days, respectively. **b** The bolting time of the *Atbhlh112* mutant, Col-0, and two *35S::OsbHLH068* transformants grown under LD (*left*) and SD (*right*) conditions. The values are the mean ± SD of two independent experiments, each with ten replicates. *P < 0.05; **P < 0.01, Student’s *t*-test. **c** Histochemical staining of *OsbHLH068p::GUS* transgenic *Arabidopsis* aerial tissues during the vegetative, transitional, and reproductive stages. Seedlings were grown under LD conditions.* Scale bar*, 1 cm. Enlarged versions of each black-disc-labeled image are shown in Supplementary Fig. S5a. **d** Histochemical staining of dark- and LD-treated *OsbHLH068p::GUS* transgenic aerial tissues over time. Seedlings were grown under LD conditions for 11 days and then transferred to darkness or constant LD conditions for an additional 3 days.* Scale bar*, 1 cm. Enlarged versions of each *black disc-labeled* image are shown in Supplementary Fig. S6. *Atbhlh112* mutant, SALK_148540

Because OsbHLH068 exerts a pleiotropic effect on many aspects of growth and development in *Arabidopsis* during the vegetative stage, we subsequently investigated whether *OsbHLH068*-overexpressing transgenic *Arabidopsis* had a different phenotype during the reproductive stage. As shown in Fig. 4a, b, the time to flowering in Col-0 was approximately 17 days when grown under LD conditions but was delayed to 21 and 19 days in the *35S::OsbHLH068* (6) and (9) transformants, respectively. Additionally, the flowering times of *35S::OsbHLH068* (6) and the (9) transformants were also delayed when grown under SD conditions (Fig. 4a, b). To validate that the delayed flowering time was not a side effect derived from the constitutive expression of *OsbHLH068* in transgenic *Arabidopsis* seedlings, the spatiotemporal expression of *OsbHLH068* was investigated by histochemical staining using *OsbHLH068::GUS* transgenic *Arabidopsis*. As shown in Fig. 4c, *GUS* was expressed in transgenic seedlings during the transition from the vegetative (14 days old) to the reproductive (17 days old) stage. GUS signals were localized only to the cotyledon and its axis in 14-day-old transgenic *Arabidopsis* seedlings but were also detected in the upper true leaves of 17-day-old transgenic seedlings (Supplementary Fig. S5A). Microscopic observation revealed that GUS signals were localized to the florets, hydathodes, leaf veins, trichome bases, and vascular bundles of the inflorescence in 17-day-old transgenic seedlings (Supplementary Fig. S5B). Because day length plays a key role in determining the flowering time of *Arabidopsis*, we further examined whether the expression of *OsbHLH068* was affected by illumination. As shown in Fig. 4d and Supplementary Fig. S6, the GUS signals in transgenic seedlings decayed when grown in the dark compared with the signals in plants grown under LD conditions. These data suggest that light-induced *OsbHLH068* plays a negative role in regulating flowering time in *Arabidopsis*. Notably, both *Atbhlh112* mutants (SALK_033618 and _148540) also displayed a late-flowering phenotype under LD conditions (Fig. 4a, b; Supplementary Fig. S7). These data suggest that OsbHLH068 and AtbHLH112 may act oppositely to control flowering time in *Arabidopsis*.

**Fig. 5:**
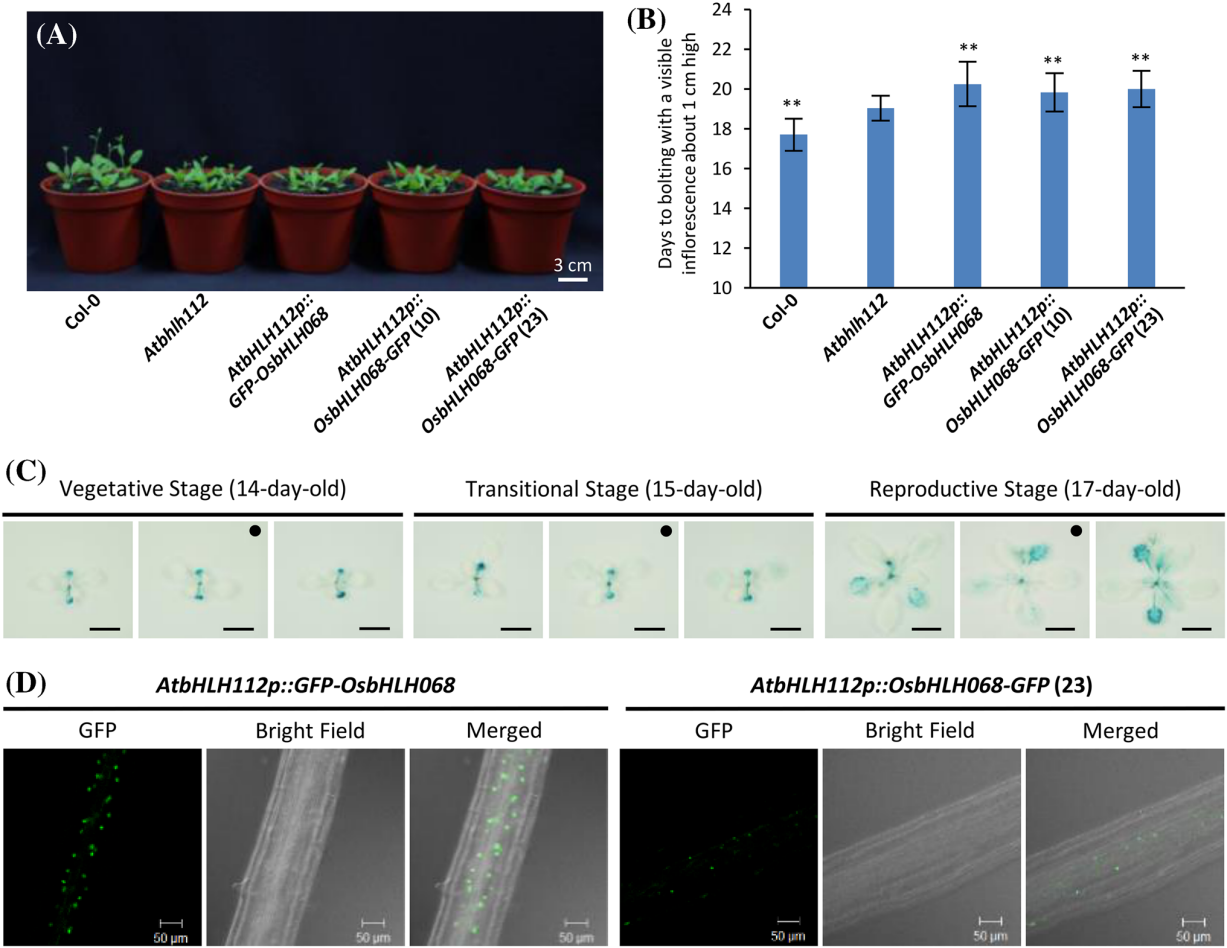
Complementary expression of *GFP-OsbHLH068* or *OsbHLH068-GFP* causes a more severe late-flowering phenotype in the *Atbhlh112* mutant background grown under LD conditions. **a** Phenotypic comparison of Col-0, the *Atbhlh112* mutant, and the three complemented transformants. The seedlings were grown in soil for 19 days. **b** The bolting time of Col-0, the *Atbhlh112* mutant, and the three complemented transformants. The values are the mean ± SD of two independent experiments, each with ten replicates. *P < 0.05; **P < 0.01, Student’s *t*-test. **c** Histochemical staining of the aerial tissues in *AtbHLH112p::GUS* transgenic seedlings during vegetative, transitional, and reproductive stages. Seedlings were grown in soil for the indicated period of time. *Scale bar* 1 cm. Enlarged versions of each *black disc-labeled* image are shown in Supplementary Fig. S8A. **d** Subcellular localization of GFP-OsbHLH068 (*left panel*) and OsbHLH068-GFP (*right panel*) proteins in complemented transformants. *Atbhlh112* mutant, SALK_148540

To confirm that OsbHLH068 and AtbHLH112 act oppositely to control flowering time, we subsequently investigated the flowering times of *Atbhlh112*, Col-0, and three complemented transformants under LD conditions. Indeed, the flowering times of the three complemented transformants were more severely delayed than *Atbhlh112* when compared to Col-0 seedlings under LD conditions (Fig. 5a, b). Additionally, *GUS* driven by a 2.2-kb *AtbHLH112* native promoter, as used in the complemented transformants expressing *GFP-OsbHLH068* or *OsbHLH068-GFP*, was expressed in the *AtbHLH112::GUS* transformant during the transition from the vegetative (14 days old) to the reproductive (17 days old) stage (Fig. 5c). Similar to *OsbHLH068::GUS* transgenic *Arabidopsis* plants, the GUS signals were localized to the cotyledon and its axis in 14-day-old *AtbHLH112::GUS* seedlings and presented in the upper true leaves of 17-day-old *AtbHLH112::GUS* seedlings (Supplementary Fig. S5A vs. S8A). Microscopic observation revealed GUS signal localization in the florets, leaf veins, and trichome bases of 17-day-old *AtbHLH112::GUS* seedlings (Supplementary Fig. S8B). Incidentally, both the GFP-OsbHLH068 and OsbHLH068-GFP fusion proteins were localized to the nucleus of root cells in the complemented transformants (Fig. 5d). Taken together, these data indicate that *OsbHLH068* and *AtbHLH112* share a similar expression pattern during the phase transition from vegetative to reproductive growth but act oppositely to control the flowering time.

### Transcriptomic analysis of OsbHLH068-overexpressing transgenic Arabidopsis plants

**Fig. 6:**
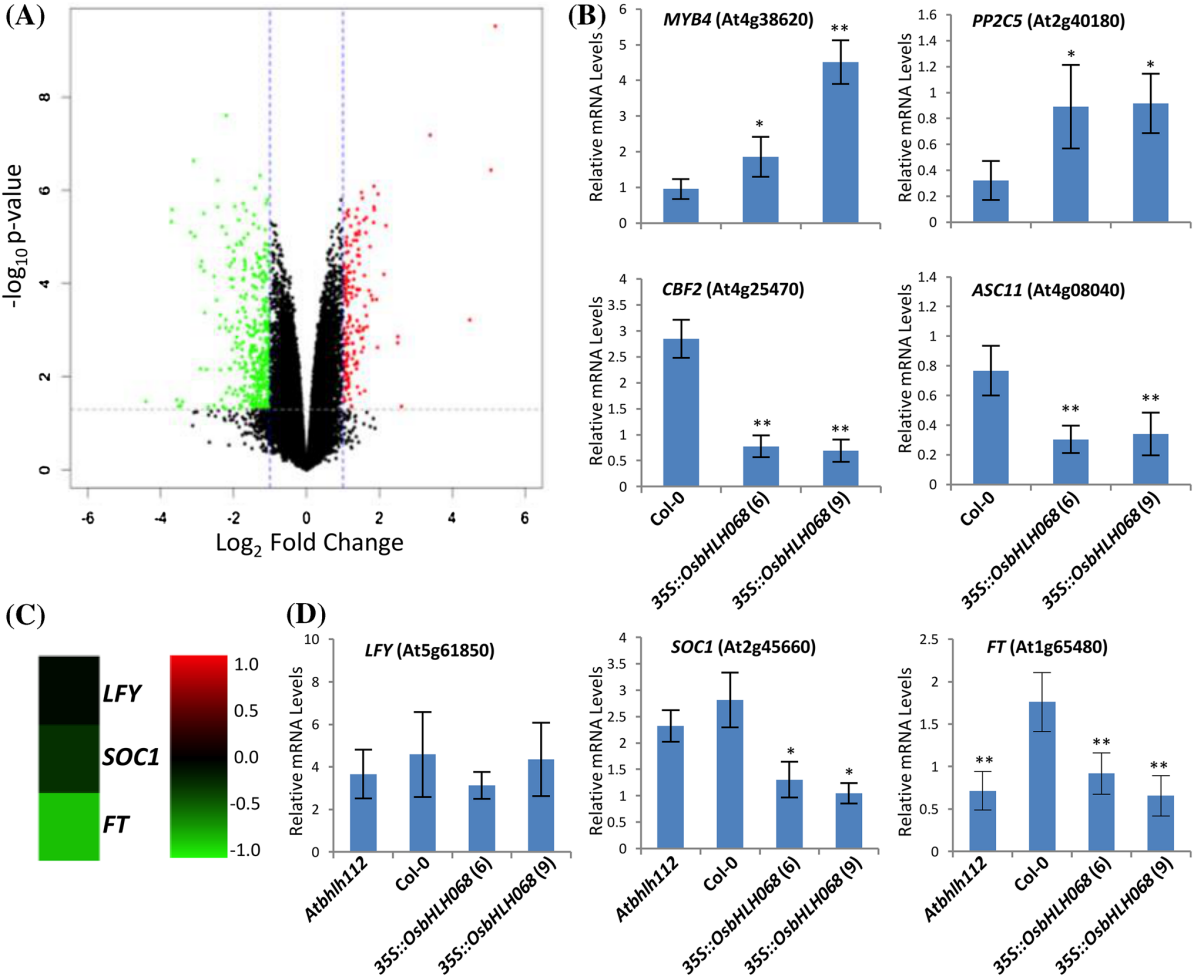
Transcriptomic analysis of the *OsbHLH068*-overexpressing transformant and Col-0. **a** Identification of DEPs. *Red and green spots* indicate up- and down-regulated DEPs, respectively. OsbHLH068-related DEPs are documented in Supplementary Data 1. **b** Quantification of *MYB4, PP2C5, CBF2*, and *ACS11* mRNAs in two *OsbHLH068*-overexpressing transgenic *Arabidopsis* lines and Col-0 by qPCR. **c** Microarray expression analysis of *LFY, SOC1*, and *FT* in *OsbHLH068*-overexpressing transgenic *Arabidopsis* plants. The *scale bar* shows log_2_-fold changes. *Red and green* colors indicate increased and decreased gene expression. **d** Quantification of *LFY, SOC1*, and *FT* mRNAs in *Atbhlh112*, Col-0, and two *OsbHLH068*-overexpressing transgenic *Arabidopsis* lines by qPCR. The total RNA used in the microarray and qPCR assays was extracted from the same aerial tissues of 17-day-old seedlings. The values presented in **b** and **d** are the mean ± SE of 4 biological replicates, each with two technical replicates. *P < 0.05; **P < 0.01, Student’s *t*-test. *Atbhlh112* mutant, SALK_148540

To better understand the molecular mechanism of *OsbHLH068* in the regulation of the salt stress response and flowering, we conducted a comparative transcriptomic analysis of *OsbHLH068*-overexpressing transformant [*35S::OsbHLH068* (9)] and Col-0 seedlings. After background correction and normalization, a total of 568 differentially expressed probes (DEPs) were identified. Among these, 168 DEPs were up-regulated while 400 DEPs were down-regulated in *OsbHLH068*-overexpressing transformants (Fig. 6a; Supplementary Data 1). As expected, several well-known stress-responsive genes were found among these DEPs, including *MYB domain protein 4* (*MYB4*), *protein phosphatase 2 C 5* (*PP2C5*), *CBF2*, and *1-aminocyclopropane-1-carboxylate synthase 11* (*ACS11*). Notably, almost none of the flowering-related genes, such as *LFY, SOC1*, and *FT*, were detected as DEPs. However, the log_2_-fold changes in *SOC1* and *FT* were approximately −0.25 and −0.79, respectively, with a *P-value* of at least < 0.05 based on Student’s *t*-test (Fig. 6c, Supplementary Data 1). Thus, a qPCR assay was performed to verify the microarray data. As shown in Fig. 6b, d, the mRNA levels of *MYB4* and *PP2C5* were higher in *OsbHLH068*-overexpressing transformants than in Col-0, while the mRNA levels of *CBF2, ACS11, SOC1*, and *FT* were lower in *OsbHLH068*-overexpressing transformants than in Col-0. The results obtained by qPCR were consistent with the microarray data. In fact, the qPCR data also revealed that the late-flowering *Atbhlh112* mutant had a relatively low level of both *FT* and *APETALA1* (*AP1*) expression and a relatively high level of *FLOWERING LOCUS C* (*FLC*) expression, which were similar to those seen in the *OsbHLH068*-overexpressing transformants (Fig. 6d; Supplementary Fig. S9).

### Common differentially expressed genes between OsbHLH068- and AtbHLH112-overexpressing Arabidopsis

**Fig. 7:**
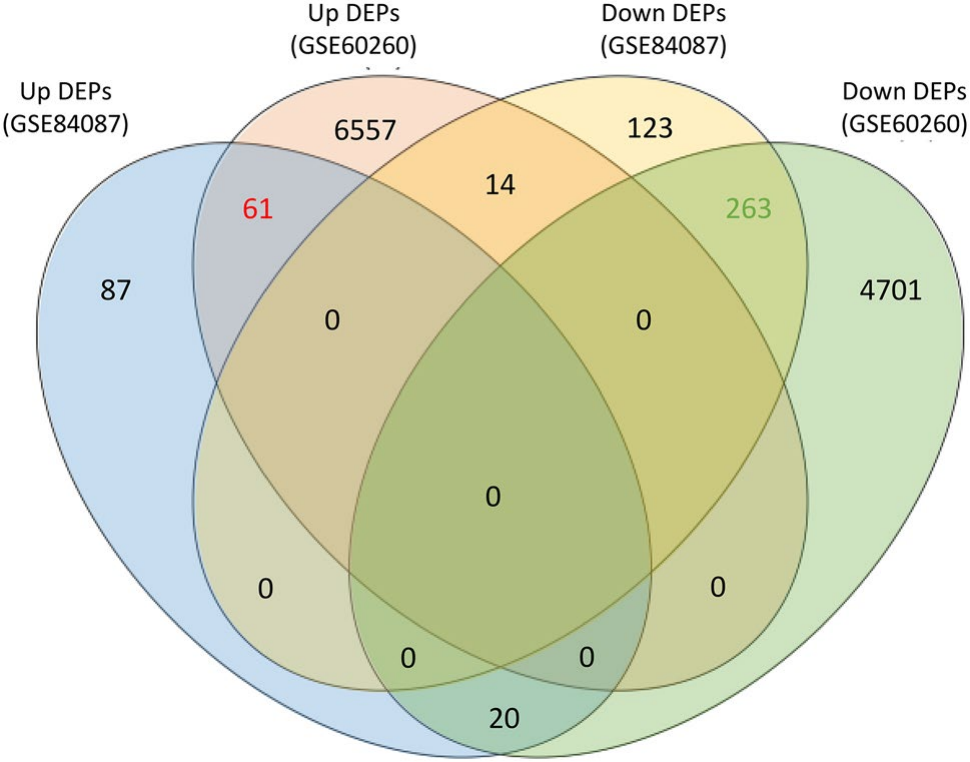
Venn diagram analysis of the DEPs between *OsbHLH068*- and *AtbHLH112*-overexpressing transgenic *Arabidopsis* plants

Because OsbHLH068 and AtbHLH112 share a highly similar bHLH domain, we assumed that both TFs should commonly regulate the expression of certain downstream genes, especially when they were constitutively expressed in the same genetic background. Therefore, we further comparatively analyzed the publicly available microarray data, GSE84087 and GSE60260, to identify the common targets between *OsbHLH068*- and *AtbHLH112*-overexpressing transgenic *Arabidopsis*. After background correction and normalization of *AtbHLH112*-related microarray data (GSE60260), a total of 11,616 DEPs consisting of 6632 up-regulated and 4984 down-regulated DEPs were identified (Supplementary Data 2). Notably, a total of 358 DEPs were commonly presented in both *OsbHLH068*- and *AtbHLH112*-overexpressing transgenic *Arabidopsis* (Fig. 7), of which 61 and 263 DEPs were identically up- and down-regulated, respectively, in both transgenic plants. Additionally, the 61 common up-regulated DEPs can be reflected in 52 genes (denoted as common up-regulated DEGs) based on TIGR annotation, while the 263 common down-regulated DEPs can be represented in 206 genes (designated as common down-regulated DEGs), including *CBF2* and *ACS11* (Supplementary Data 3). Gene ontology analysis revealed that the major ontological categories of the commonly regulated DEGs were ‘response to stimulus’ and ‘transcription regulator activity’ of ‘biological process’ and ‘molecular function’, respectively (Supplementary Fig. S10). Incidentally, further promoter analysis indicated that 51 and 194 common up- and down-regulated DEGs, respectively, contain at least one E-box element in their 1-kb promoter region (Supplementary Data 4).

## Discussion

### Partial functional redundancy of AtbHLH112 and OsbHLH068 in the stress response

The phytohormone abscisic acid (ABA) plays an important role in regulating seed maturation, dormancy, stomatal closure, and abiotic stress responses (Gubler et al. 2005; Karssen et al. 1983; Leung and Giraudat 1998; MacRobbie 1998; Seiler et al. 2011). In fact, ABA-mediated seed dormancy is an adaptive mechanism that maintains viable seeds in a quiescent state and leads to the escape from or avoidance of a stressful environment (Seo et al. 2006). Indeed, overexpression of ABA biosynthetic genes, such as *nine-cis-epoxycarotenoid dioxygenase 3* (*AtNCED3*) and *ABA2*, delays seed germination and confers drought/salt stress tolerance in transgenic *Arabidopsis* plants (Cheng et al. 2002; Iuchi et al. 2001; Lin et al. 2007). In contrast, ABA-deficient mutants, such as *aba1, aba2*, and *aba4*, have a rapid seed germination phenotype but are susceptible to osmotic stress (Lin et al. 2007; North et al. 2007). Additionally, root architecture alteration is another adaptive mechanism for plants to survive under stress conditions (Price et al. 1997; Serraj et al. 2004; Uga et al. 2013). In rice, drought-resistant varieties usually develop a deeper root system so that the roots can take up water from deeper soil layers to manage water deficiency (Gowda et al. 2011; Price et al. 1997; Uga et al. 2013). As mentioned in a previous study, AtbHLH112 confers abiotic stress tolerance by enhancing the ROS scavenging ability and promotes primary root growth under salt-treated conditions (Liu et al. 2015). In this study, heterologous overexpression of *OsbHLH068*, an *AtbHLH112* homolog, in *Arabidopsis* delayed seed germination, decreased the accumulation of MDA and H_2_O_2_, and enhanced primary root elongation under salt-treated conditions, whereas the *Atbhlh112* mutant displayed a rapid seed germination phenotype, a relatively high level of MDA and H_2_O_2_, and a short root length phenotype (Fig. 3a–c and Supplementary Fig. S4). Notably, the seed germination and root elongation properties of *OsbHLH068*-overexpressing transgenic *Arabidopsis* plants and the *Atbhlh112* mutant were highly similar to those of the ABA over-accumulating *35S::LfNCED3* transformant and the ABA-deficient mutant, *Atnced3*, respectively. Furthermore, complementary expression of either *GFP-OsbHLH068* or *OsbHLH068-GFP* driven by a 2.2-kb *AtbHLH112* promoter could restore, partially or completely, the early germination and the H_2_O_2_ over-accumulation of the *Atbhlh112* mutant to normal germination and accumulation, respectively (Fig. 3a, b). These data showed that the regulatory role of AtbHLH112 in seed germination and H_2_O_2_ scavenging could be replaced by OsbHLH068, presumably due to the conserved function between *Arabidopsis* (dicot) and rice (monocot). Additionally, comparative transcriptomic analysis indicated that 52 up-regulated and 206 down-regulated DEGs were commonly presented in *OsbHLH068*- and *AtbHLH112*-overexpressing transgenic *Arabidopsis* plants (Supplementary Data 3), of which 51 common up-regulated DEGs and 194 common down-regulated DEGs had at least one E-box element (5′-CANNTG-3′) in their 1-kb promoter region, including several well-known stress-responsive genes [e.g., *AtERF15* (At2g31230), *AtPUB23* (At2g35930) and *WRKY48* (At5g49520)] (Supplementary Data 4). *AtERF15*, a commonly up-regulated DEG, plays a positive role in regulating ABA-mediated drought tolerance, whereas *plant U-box 23* (*AtPUB23*), a commonly down-regulated DEG, negatively regulates ABA-mediated drought stress responses (Lee et al. 2015; Seo et al. 2012). The osmotic stress- and pathogen-induced *AtWRKY48*, a common down-regulated DEG, functions as a negative regulator of defense-related genes, such as *PRs*, and basal resistance to the bacterial pathogen *Pseudomonas syringae* (Xing et al. 2008). Taken together, our data reveal that *OsbHLH068* plays a role similar to that of *AtbHLH112* in the regulation of abiotic stress responses, presumably due to the partially functional redundancy of these homologous genes.

### Functional divergence of AtbHLH112 and OsbHLH068 in flowering control

Homologous gene-encoded proteins usually play a similar role in regulating plant developmental and physiological processes. For example, the CBFs/DREBs, which belong to the AP2/ERF family, play a positive regulatory role in the cold tolerance of evolutionarily diverse plant species, including *Arabidopsis* and rice (for a review, see Chinnusamy et al. 2010). Notably, both OsbHLH068 and AtbHLH112 are members of the F subfamily (Li et al. 2006). In the F subfamily, both the OsbHLH068 domain and the AtbHLH112 domain share a high sequence similarity (Supplementary Fig. S1). Additionally, *OsbHLH068* and *AtbHLH112* are expressed in the upper true leaves of transgenic *Arabidopsis* seedlings in a nearly overlapping pattern during the juvenile-to-adult phase (Fig. 4c vs. 5c, Supplementary Fig. S5 vs. S8). These biological characteristics suggest that OsbHLH068 and AtbHLH112 should play similar roles in regulating plant growth and development, especially during the phase transition process. Indeed, heterologous overexpression of *OsbHLH068* in *Arabidopsis* delayed flowering with a relatively low level of *SOC1, FT*, or *AP1* expression and a relatively high level of *FLC* expression under LD conditions (Figs. 4a, b, 6c, d; Supplementary Fig. S9). Surprisingly, two *Atbhlh112* mutant lines (SALK_033618 and _148540) also displayed a late-flowering phenotype under LD conditions (Fig. 4a, b; Supplementary Fig. S7). Interestingly, the late-flowering *Atbhlh112* mutant (SALK_148540) exhibited a relatively low level of both *FT* and *AP1* expression and a relatively high level of *FLC* expression under LD conditions, whereas constitutive expression of *AtbHLH112* in *Arabidopsis* up-regulated the expression of *SOC1* but not *FT* (Fig. 6d; Supplementary Fig. S9 and Data 2). Inconceivably, complementary expression of *GFP-OsbHLH068* or *OSbHLH068-GFP* driven by a 2.2-kb *AtbHLH112* promoter in the late-flowering *Atbhlh112* mutant delayed the flowering time more severely under LD conditions (Fig. 5a, b). The opposite effect of *OsbHLH068* and *AtbHLH112* on flowering control did not appear to be caused by the differential transcriptional activity because complementary expression of *GFP-OsbHLH068* or *OsbHLH068-GFP* driven by a 2.2-kb *AtbHLH112* promoter in the *Atbhlh112* mutant partially or completely restored the early seed germination and H_2_O_2_ over-accumulation to normal levels (Fig. 3a, c). Therefore, we assume that OsbHLH068 and AtbHLH112 act oppositely in flowering control, presumably due to the divergent evolution of plant flowering.

**Fig. 8:**
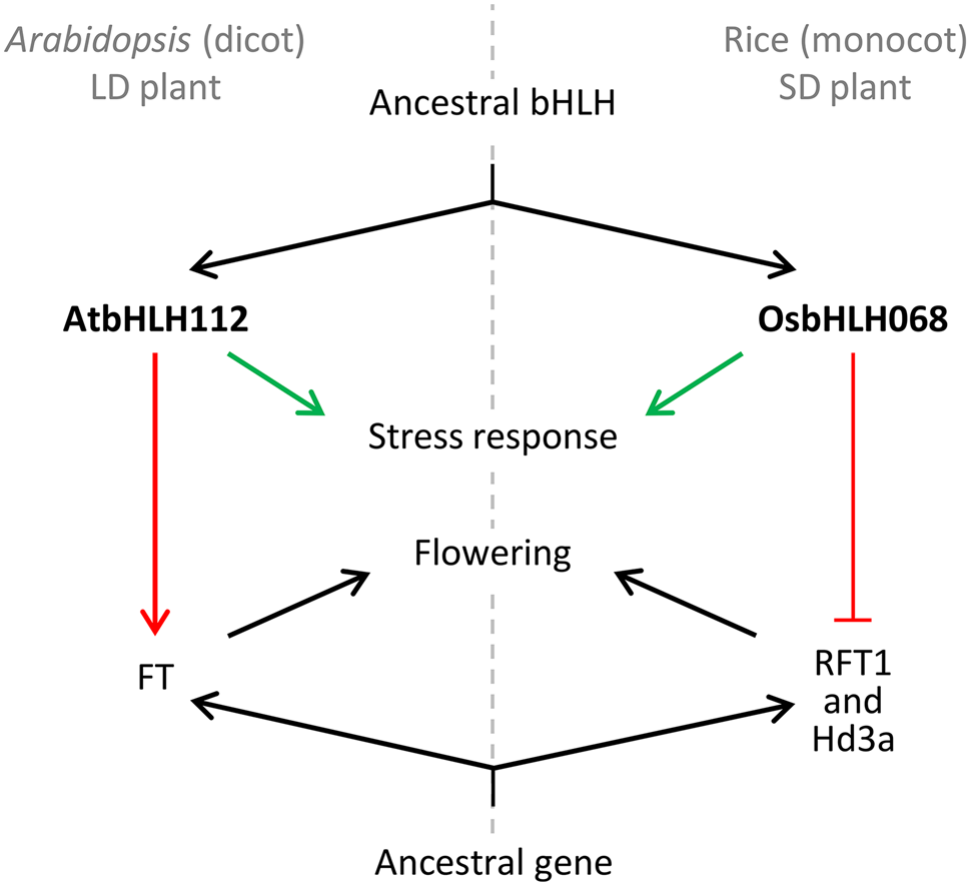
Schematic diagram of the functional redundancy and functional divergence in the homologous AtbHLH112- and OsbHLH068-mediated stress response and flowering. *Green and red arrows* indicate functional redundancy and functional divergence of homologous AtbHLH112- and OsbHLH068, respectively

Divergent evolution is a pathway involved in speciation that contributes to the species abundance and diversity of biological systems. Gene duplication, followed by the functional divergence of paralog-encoded proteins, is considered to be a major driving force in the evolution of biological diversity (for review, see Kondrashov et al. 2002; Ohno 1970). For example, *ATX1* and *ATX2*, two *Arabidopsis thaliana TRITHORAX* homologous genes derived from a segmental duplication, belong to the same clade as the sister paralogs (Alvarez-Venegas and Avramova 2002; Baumbusch et al. 2001). Although the structure of *ATX1* is similar to that of *ATX2*, ATX1 and ATX2 have opposite biochemical activities (Saleh et al. 2008): ATX1 trimethylates K4 of histone H3, whereas ATX2 dimethylates it. They activate and inactivate the transcriptional expression of common target genes, including *WRKY70*, a TF gene involved in the regulation of the disease response. In addition to *ATX1* and *ATX2*, the *ARABIDOPSIS MYOTUBULARIN1* (*AtMTM1*) and *AtMTM2* homologs also originated from a segmental duplication and encode catalytically active enzymes with a similar domain architecture and a conserved biochemically active catalytic site (Ding et al. 2012). However, AtMTM1 elevates the cellular level of phosphatidylinositol 5-phosphate (PtdIns5P) in response to dehydration stress, but the function of AtMTM2 remains unclear. More importantly, AtMTM1-mediated PtdIns5P can bind to the PHD domain of ATX1 and then repress the methylation activity of ATX1 (Alvarez-Venegas et al. 2006a, b; Ndamukong et al. 2010). These cases illustrate that the functional divergence of paralog-encoded proteins confers, at least in part, the biochemical and biological diversity of plants. To identify flowering-related genes in monocot rice, a facultative SD plant, the transcriptional expression of two *FT* homologous genes, *Hd3a* and *RFT1* paralogs, is activated by Ehd1 in the vascular tissues of leaf blades under both SDs and LDs (Doi et al. 2004). However, the transcriptional expression of *Hd3a* is repressed by Hd1, a CO homolog, under LDs (Kojima et al. 2002). Notably, no *Ehd1* homolog was found in *Arabidopsis*. In contrast, the dicot *Arabidopsis* is classified as a facultative LD plant. Transcriptional expression of *FT* is activated by CO in the vascular tissues of leaf blades under LDs (Kardailsky et al. 1999). Interestingly, the transcriptional regulation of homologous *Hd3a* and *FT* by Hd1 and CO, two homologous zinc-finger TFs, also displays an opposite relationship in the flowering control between rice and *Arabidopsis*, respectively, under LDs. These data indicate that functional divergence of homologous flowering-related genes was present during the divergent evolution of rice and *Arabidopsis*. The present data indicate that *OsbHLH068* and *AtbHLH112* have a similar transcriptional pattern during the juvenile-to-adult phase transition, but both *OsbHLH068*-overexpressing transgenic *Arabidopsis* plants and the *Atbhlh112* mutants show a late-flowering phenotype with a relatively low level of *FT* expression under LDs. Notably, constitutive expression of *AtbHLH112* in transgenic *Arabidopsis* seedlings resulted in a relatively high expression level of *SOC1* but no difference from that of *FT* (Supplementary Data 2). Further promoter analysis revealed that at least 5, 3, 4, and 6 E-box elements were located in the promoter region within 1 kb upstream of the *FT, SOC1, Hd3a*, and *RFT* transcription start sites, respectively (Supplementary Table 1). Thus, we conclude that OsbHLH068 and AtbHLH112, two homologous TFs, have an opposite effect on the transcriptional regulation of downstream homologous flowering genes between rice and *Arabidopsis*, which is similar to the effect observed for Hd1 and CO in the transcriptional regulation of *Hd3a* and *FT*, respectively. Additionally, AtbHLH112 seems to play a fine-tuning role in regulating flowering time by interacting with the different proteins in *Arabidopsis*. Taken together, we propose that the opposite roles of OsbHLH068 and AtbHLH112 in flowering control, as well as *Hd1* and *CO*, are to be at least partially incorporated into the divergent evolution of rice and *Arabidopsis* (for a summary, see Fig. 8).

### A putative role of OsbHLH068 in salt stress response and flowering

Flowering is important for plants to complete the life cycle. However, the timing of flowering is highly susceptible to environmental conditions, particularly when the plant is exposed to abiotic stresses. In fact, many studies have shown that the effects of abiotic stress on flowering control are different in various plant species. For example, the flowering of *Arabidopsis* plants is accelerated by drought and delayed by salinity (Kazan and Lyons 2016; Riboni et al. 2014), whereas the flowering of rice plants is delayed by drought (Galbiati et al. 2016). Notably, previous studies have also shown that certain stress-inducible TFs play important roles in connecting environmental factors to flowering, including *CmMYB2* and *OsABF1*. Heterologous overexpression of *CmMYB2*, an abiotic stress-inducible R2R3-MYB TF in chrysanthemum, improves osmotic-stress tolerance and delays flowering time in transgenic *Arabidopsis* plants (Shan et al. 2012). In rice, a drought-inducible bZIP TF, *OsABF1*, acts as a negative regulator of floral transition upon water deficit (Zhang et al. 2016). In this study, our data reveal that heterologous overexpression of *OsbHLH068*, a salt-inducible TF, in *Arabidopsis* results in decreased salt-dependent accumulation of either MDA or H_2_O_2_, increased root length, and delayed flowering. In fact, the *OsbHLH068*-overexpressing transgenic rice plants also displayed a late-flowering phenotype with a relatively low level of *Hd3a* and *RFT1* expression (unpublished data). Thus, we speculate that *OsbHLH068* may play a pivotal role in linking the salt stress response to rice flowering.

## Materials and methods

### Plant materials and growth conditions

In *Arabidopsis*, the SALK_033618 and _148540 lines are *AtbHLH112* knock-out mutants, which have been documented in a previous study (Wang et al. 2014). These mutants were obtained from the *Arabidopsis* Biological Resource Center (ABRC, OH). All mutants were derived from the Columbia-0 (Col-0) accession. Seeds were treated with 1.2% (v/v) commercial bleach for 15 min, rinsed twice with sterile water for 10 min each, and subsequently stored at 4 °C in the dark for 3 days. Sterilized and cold-pretreated seeds were sown on agar plates or in pots containing disinfected Tref substrate (Jiffy). Seedlings were grown at 24 °C under LD (16-h light/8-h dark cycle) or SD (8-h light/16-h dark cycle) conditions with a light intensity of approximately 100 µE/s m^2^. For the germination and root elongation tests, the basal agar medium was composed of half-strength MS salts (Murashige and Skoog 1962), B5 organic compounds (Gamborg et al. 1968), 0.05% MES [2-(N-morpholino)ethane sulfonic acid monohydrate], and 1% sucrose. The *Atnced3* mutant and *35S::LfNCED3* transformant were obtained from Dr. Wan-Hsing Cheng (Institute of Plant and Microbial Biology, Academia Sinica, Taipei, Taiwan) and have been used in previous studies (Chen et al. 2011; Wan and Li 2006).

In rice, *Oryza sativa* L. cv. Nipponbare and *Oryza sativa* L. cv. Tainung 67 were investigated in the present analysis. The seeds were sterilized and imbibed at 37 °C for 2 days in the dark. After imbibition, the germinated seeds were grown on a wired stand in beakers at 28 °C under LD conditions (16-h light/8-h dark cycle) with a light intensity of approximately 100 µE/s m^2^. The basal medium was Kimura B solution (Yoshida et al. 1976).

### Transgene constructs

The full-length coding sequences of *OsbHLH068* and *GFP*, with or without a stop codon, were PCR-amplified and cloned into the pGEM-T Easy vector (Promega). These fragments were then subcloned into a binary vector, pCAMBIA-1300, where their expression was driven by either a 2.2-kb *AtbHLH112* native promoter (*AtbHLH112p::GFP-OsbHLH068* and *AtbHLH112p::OsbHLH068-GFP*) or a CaMV 35 S promoter (*35S::OsbHLH068*). After sequencing, the *AtbHLH112p::GFP-OsbHLH068* and *AtbHLH112p::OsbHLH068-GFP* constructs were subsequently transformed into the *Atbhlh112* (SALK_148540) mutant by the floral dipping method for functional complementation and protein subcellular localization assays, and the *35S::OsbHLH068* construct was introduced into Columbia-0 for functional analysis. Additionally, both 1.2-kb *OsbHLH068* and 2.2-kb *AtbHLH112* native promoters were used to replace the 35 S promoter to drive the expression of the β-glucuronidase (GUS) reporter gene in the binary vector pCAMBIA-1305.1. After sequencing, the *OsbHLH068p::GUS* and *AtbHLH112p::GUS* constructs were subsequently transformed into Col-0 and/or *Oryza sativa* L. cv. Tainung 67 via an *Agrobacterium*-mediated method for analyzing spatiotemporal gene expression.

### Phenotypic comparisons and plant weight measurements

For the phenotypic comparisons, cold-pretreated seeds from each type of *Arabidopsis* plant were grown in pots containing disinfected Tref substrate (Jiffy). For the plant weight measurements, seedlings were grown under LD conditions. The aerial parts of the 15-day-old plants were excised and the detached rosette leaves were used to measure the fresh weight. These detached tissues were subsequently vacuum dried at least overnight to measure the dry weight. All fresh and dry weights are the mean ± SD of two independent experiments, each with 6 biological replicates (each with an average value of two technical replicates).

### Germination and root elongation tests

For the germination and root elongation tests, cold-pretreated seeds of each type of *Arabidopsis* plant were sown on agar plates containing basal agar medium with or without 100 mM NaCl. Germination was defined as the point when radicle emergence first started to exceed seed coat. All germination rates are the mean ± SE of 4 independent biological replicates. Each biological replicate contains at least 35 seeds that were harvested from an individual *Arabidopsis* plant. The root length was measured after vertical growth for 5 days on agar plates. All root lengths are the mean ± SE of 20 independent biological replicates.

### MDA and H_2_O_2_ determinations

For the MDA and H_2_O_2_ assays, cold-pretreated seeds of each type of *Arabidopsis* plant were grown in pots containing disinfected Tref substrate (Jiffy) under LD conditions. After growing for 12 days in pots, the seedlings were watered with fresh water containing 0 or 250 mM NaCl for an additional 3 days and then harvested for analysis. The MDA and H_2_O_2_ levels were measured according to methods described by Heath and Packer (1968), Jana and Choudhuri (1981), respectively.

### RNA extraction and cDNA synthesis

Total RNA was extracted from the various plant tissues using an RNeasy Mini Kit (Qiagen) according to the manufacturer’s instructions. To avoid genomic DNA contamination, total RNA was treated with Turbo DNA-*free*™ DNase (Ambion) following the manufacturer’s instructions. The DNase-treated total RNA was subjected to cDNA synthesis using the SuperScript™ III first-strand synthesis system (Invitrogen) according to the manufacturer’s instructions.

### Reverse transcription-PCR (RT-PCR) and quantitative PCR (qPCR)

The initial amount of template cDNA in each RT-PCR and qPCR reaction was 25 and 10 ng, respectively. The qPCR reactions were performed using an ABI 7500 system with the SYBR^®^ Green PCR Master Mix Kit [Applied Biosystems (ABI)]. The 2^−ΔCT^ was used to show a difference between the target gene and internal control. *OsACTIN1* was used as an internal control for qPCR normalization. The primer sequences used are listed in Supplementary Table 2.

### Spatiotemporal gene expression and protein subcellular localization

Samples were soaked in fixation buffer (0.3% formaldehyde, 10 mM MES hydrate, 0.3 M mannitol, and 2 mM dithiothreitol, pH 5.6) for 30 min before staining. After fixation, the samples were rinsed twice with 50 mM sodium phosphate (pH 7.0) and then submerged in a staining solution [50 mM sodium phosphate dibasic, 0.5 mM potassium ferrocyanide, 0.5 mM potassium ferricyanide, 2 mM dithiothreitol, and 1 mM X-Gluc (5-bromo-4-chloro-3-indolyl β-D-glucuronide cyclohexylammonium salt), pH 7.0]. The staining assay was conducted under ambient conditions. For the protein subcellular localization assay, GFP fluorescence was detected with spectral settings of 500–540 nm for emission and 488 nm for excitation using a Zeiss LSM 510 Meta confocal microscope.

### Microarray and data analysis

Total RNA was amplified and labeled using a Low-Input Quick Amp Labeling Kit, One-Color (Agilent, USA), according to the manufacturer’s instructions. Cyanine 3 (Cy3)-labeled cRNA was fragmented by incubation at 60 °C for 30 min. After fragmentation, Cy3-labeled cRNA was pooled and hybridized to the Agilent *Arabidopsis* V4 Oligo 4 × 44 K Microarray as suggested by the manufacturer. The array image was analyzed using Feature Extraction software version 10.7.1.1 with default settings. The gene expression data are available under accession number GSE84087. Additionally, the microarray dataset (GSE60260) was obtained from the Gene Expression Omnibus website (http://www.ncbi.nlm.nih.gov/geo/). To identify DEPs, the raw microarray data were analyzed with the Bioconductor Limma package (Ritchie et al. 2015). The raw data were background-corrected using the ‘normexp’ method and then normalized using the ‘quantile’ method. Each up- or down-regulated DEP had a log_2_-fold change > or <1, respectively, with a *P-value* < 0. 05 based on Student’s *t*-test. Each DEP-annotated gene is listed and described in the corresponding supplemental data. Common DEG ontology graphical analysis was conducted using the agriGO database (Du et al. 2010).

## Electronic supplementary material

Below is the link to the electronic supplementary material.

Supplementary material 1 (PDF 1762 KB)

Supplementary material 2 (PDF 815 KB)

Supplementary material 3 (PDF 173 KB)

Supplementary material 4 (XLSX 13115 KB)

Supplementary material 5 (XLSX 19148 KB)

Supplementary material 6 (XLSX 297 KB)

Supplementary material 7 (XLSX 41 KB)

## References

[CR1] Alvarez-Venegas R, Avramova Z (2002). SET-domain proteins of the Su(var)3–9, E(z) and Trithorax families. Gene.

[CR2] Alvarez-Venegas R, Sadder M, Hlavacka A, Baluska F, Xia Y, Lu G, Firsov A, Sarath G, Moriyama H, Dubrovsky JG, Avramova Z (2006). The *Arabidopsis* homolog of trithorax, ATX1, binds phosphatidylinositol 5-phosphate, and the two regulate a common set of target genes. Proc Natl Acad Sci U S A.

[CR3] Alvarez-Venegas R, Xia Y, Lu G, Avramova Z (2006). Phosphoinositide 5-phosphate and phosphoinositide 4-phosphate trigger distinct specific responses of *Arabidopsis* genes. Plant Signal Behav.

[CR4] Andrés F, Coupland G (2012). The genetic basis of flowering responses to seasonal cues. Nat Rev Genet.

[CR5] Bailey PC, Martin C, Toledo-Ortiz G, Quail PH, Huq E, Heim MA, Jakoby M, Werber M, Weisshaar B (2003). Update on the basic helix-loop-helix transcription factor gene family in *Arabidopsis thaliana*. Plant Cell.

[CR6] Baumbusch LO, Thorstensen T, Krauss V, Fischer A, Naumann K, Assalkhou R, Schulz I, Reuter G, Aalen RB (2001). The *Arabidopsis thaliana* genome contains at least 29 active genes encoding SET domain proteins that can be assigned to four evolutionarily conserved classes. Nucleic Acids Res.

[CR7] Chen HC, Hwang SG, Chen SM, Shii CT, Cheng WH (2011). ABA-mediated heterophylly is regulated by differential expression of 9-cis-epoxycarotenoid dioxygenase 3 in lilies. Plant Cell Physiol.

[CR8] Cheng W-H, Endo A, Zhou L, Penney J, Chen H-C, Arroyo A, Leon P, Nambara E, Asami T, Seo M (2002). A unique short-chain dehydrogenase/reductase in *Arabidopsis* glucose signaling and abscisic acid biosynthesis and functions. Plant Cell.

[CR9] Chinnusamy V, Ohta M, Kanrar S, Lee B-H, Hong X, Agarwal M, Zhu J-K (2003). ICE1: a regulator of cold-induced transcriptome and freezing tolerance in *Arabidopsis*. Genes Dev.

[CR10] Chinnusamy V, Zhu J-K, Sunkar R (2010). Gene regulation during cold stress acclimation in plants. Methods in Mol Biol.

[CR11] Cui J, You C, Zhu E, Huang Q, Ma H, Chang F (2016). Feedback regulation of DYT1 by interactions with downstream bHLH factors promotes DYT1 nuclear localization and anther development. Plant Cell.

[CR12] Ding W, Yu Z, Tong Y, Huang W, Chen H, Wu P (2009). A transcription factor with a bHLH domain regulates root hair development in rice. Cell Res.

[CR13] Ding Y, Ndamukong I, Zhao Y, Xia Y, Riethoven J-J, Jones DR, Divecha N, Avramova Z (2012). Divergent functions of the myotubularin (MTM) homologs AtMTM1 and AtMTM2 in *Arabidopsis thaliana*: evolution of the plant MTM family. Plant J.

[CR14] Doi K, Izawa T, Fuse T, Yamanouchi U, Kubo T, Shimatani Z, Yano M, Yoshimura A (2004). Ehd1, a B-type response regulator in rice, confers short-day promotion of flowering and controls FT-like gene expression independently of Hd1. Genes Dev.

[CR15] Du Z, Zhou X, Ling Y, Zhang Z, Su Z (2010). agriGO: a GO analysis toolkit for the agricultural community. Nucleic Acids Res.

[CR16] Feller A, Machemer K, Braun EL, Grotewold E (2011). Evolutionary and comparative analysis of MYB and bHLH plant transcription factors. Plant J.

[CR17] Galbiati F, Chiozzotto R, Locatelli F, Spada A, Genga A, Fornara F (2016). *Hd3a, RFT1* and *Ehd1* integrate photoperiodic and drought stress signals to delay the floral transition in rice. Plant Cell Environ.

[CR18] Gamborg OL, Miller RA, Ojima K (1968). Nutrient requirements of suspension cultures of soybean root cells. Exp Cell Res.

[CR19] Gowda VRP, Henry A, Yamauchi A, Shashidhar HE, Serraj R (2011). Root biology and genetic improvement for drought avoidance in rice. Field Crops Res.

[CR20] Gubler F, Millar AA, Jacobsen JV (2005). Dormancy release, ABA and pre-harvest sprouting. Curr Opin Plant Biol.

[CR21] Hayama R, Yokoi S, Tamaki S, Yano M, Shimamoto K (2003). Adaptation of photoperiodic control pathways produces short-day flowering in rice. Nature.

[CR22] Heang D, Sassa H (2012). Antagonistic actions of HLH/bHLH proteins are involved in grain length and weight in rice. PLoS One.

[CR23] Heath RL, Packer L (1968). Photoperoxidation in isolated chloroplasts. I. Kinetics and stoichiometry of fatty acid peroxidation. Arch Biochem Biophys.

[CR24] Heim MA, Jakoby M, Werber M, Martin C, Weisshaar B, Bailey PC (2003). The basic helix–loop–helix transcription factor family in plants: a genome-wide study of protein structure and functional diversity. Mol Biol Evol.

[CR25] Ishikawa R, Aoki M, Kurotani K-I, Yokoi S, Shinomura T, Takano M, Shimamoto K (2011). Phytochrome B regulates heading date 1 (Hd1)-mediated expression of rice florigen Hd3a and critical day length in rice. Mol Genet Genomics.

[CR26] Ito Y, Katsura K, Maruyama K, Taji T, Kobayashi M, Seki M, Shinozaki K, Yamaguchi-Shinozaki K (2006). Functional analysis of rice DREB1/CBF-type transcription factors involved in cold-responsive gene expression in transgenic rice. Plant Cell Physiol.

[CR27] Ito S, Song YH, Josephson-Day AR, Miller RJ, Breton G, Olmstead RG, Imaizumi T (2012). FLOWERING BHLH transcriptional activators control expression of the photoperiodic flowering regulator CONSTANS in *Arabidopsis*. Proc Natl Acad Sci.

[CR28] Iuchi S, Kobayashi M, Taji T, Naramoto M, Seki M, Kato T, Tabata S, Kakubari Y, Yamaguchi-Shinozaki K, Shinozaki K (2001). Regulation of drought tolerance by gene manipulation of 9-cis-epoxycarotenoid dioxygenase, a key enzyme in abscisic acid biosynthesis in *Arabidopsis*. Plant J.

[CR29] Jana S, Choudhuri MA (1981). Glycolate metabolism of three submersed aquatic angiosperms during ageing. Aquat Bot.

[CR30] Jiang Y, Yang B, Deyholos MK (2009). Functional characterization of the *Arabidopsis* bHLH92 transcription factor in abiotic stress. Mol Genet Genomics.

[CR31] Kardailsky I, Shukla VK, Ahn JH, Dagenais N, Christensen SK, Nguyen JT, Chory J, Harrison MJ, Weigel D (1999). Activation tagging of the floral inducer FT. Science.

[CR32] Karssen CM, van der Swan DLC, Breekland AE, Koornneef M (1983). Induction of dormancy during seed development by endogenous abscisic acid: studies on abscisic acid deficient genotypes of *Arabidopsis thaliana* (L.) Heynh. Planta.

[CR33] Kazan K, Lyons R (2016). The link between flowering time and stress tolerance. J Exp Bot.

[CR34] Kojima S, Takahashi Y, Kobayashi Y, Monna L, Sasaki T, Araki T, Yano M (2002). Hd3a, a rice ortholog of the *Arabidopsis* FT gene, promotes transition to flowering downstream of Hd1 under short-day conditions. Plant Cell Physiol.

[CR35] Komiya R, Ikegami A, Tamaki S, Yokoi S, Shimamoto K (2008). Hd3a and RFT1 are essential for flowering in rice. Development.

[CR36] Komiya R, Yokoi S, Shimamoto K (2009). A gene network for long-day flowering activates RFT1 encoding a mobile flowering signal in rice. Development.

[CR37] Kondrashov FA, Rogozin IB, Wolf YI, Koonin EV (2002). Selection in the evolution of gene duplications. Genome Biol.

[CR38] Lee S-B, Lee S-J, Kim SY (2015). AtERF15 is a positive regulator of ABA response. Plant Cell Rep.

[CR39] Leung J, Giraudat J (1998). Abscisic acid signal transduction. Ann Rev Plant Physiol Plant Mol Biol.

[CR40] Li X, Duan X, Jiang H, Sun Y, Tang Y, Yuan Z, Guo J, Liang W, Chen L, Yin J, Ma H, Wang J, Zhang D (2006). Genome-wide analysis of basic/Helix-loop-helix transcription factor family in rice and *Arabidopsis*. Plant Physiol.

[CR41] Li F, Guo S, Zhao Y, Chen D, Chong K, Xu Y (2010). Overexpression of a homopeptide repeat-containing bHLH protein gene (OrbHLH001) from Dongxiang wild rice confers freezing and salt tolerance in transgenic *Arabidopsis*. Plant Cell Rep.

[CR42] Lin PC, Hwang SG, Endo A, Okamoto M, Koshiba T, Cheng WH (2007). Ectopic expression of ABSCISIC ACID 2/GLUCOSE INSENSITIVE 1 in *Arabidopsis* promotes seed dormancy and stress tolerance. Plant Physiol.

[CR43] Liu W, Tai H, Li S, Gao W, Zhao M, Xie C, Li W-X (2014). bHLH122is important for drought and osmotic stress resistance in *Arabidopsis* and in the repression of ABA catabolism. New Phytol.

[CR44] Liu Y, Ji X, Nie X, Qu M, Zheng L, Tan Z, Zhao H, Huo L, Liu S, Zhang B, Wang Y (2015). *Arabidopsis* AtbHLH112 regulates the expression of genes involved in abiotic stress tolerance by binding to their E-box and GCG-box motifs. New Phytol.

[CR45] MacRobbie EAC (1998). Signal transduction and ion channels in guard cells. Philosoph Trans Roy Soc B Biol Sci.

[CR46] Maurya JP, Sethi V, Gangappa SN, Gupta N, Chattopadhyay S (2015). Interaction of MYC2 and GBF1 results in functional antagonism in blue light-mediated *Arabidopsis* seedling development. Plant J.

[CR47] Mouradov A, Cremer F, Coupland G (2002). Control of flowering time: interacting pathways as a basis for diversity. Plant Cell.

[CR48] Murashige T, Skoog F (1962). A revised medium for rapid growth and bio assays with tobacco tissue cultures. Physiol Plant.

[CR49] Murre C, McCaw PS, Baltimore D (1989). A new DNA binding and dimerization motif in immunoglobulin enhancer binding, daughterless, MyoD, and myc proteins. Cell.

[CR50] Ndamukong I, Jones DR, Lapko H, Divecha N, Avramova Z (2010). Phosphatidylinositol 5-phosphate links dehydration stress to the activity of ARABIDOPSIS TRITHORAX-LIKE Factor ATX1. PLoS One.

[CR51] North HM, Almeida AD, Boutin J-P, Frey A, To A, Botran L, Sotta B, Marion-Poll A (2007). The *Arabidopsis* ABA-deficient mutant aba4 demonstrates that the major route for stress-induced ABA accumulation is via neoxanthin isomers. Plant J.

[CR52] Ohno S (1970). Evolution by gene duplication.

[CR53] Pires N, Dolan L (2010). Origin and diversification of basic-helix-loop-helix proteins in plants. Mol Biol Evol.

[CR54] Price AH, Tomos AD, Virk DS (1997). Erratum: genetic dissection of root growth in rice (*Oryza sativa* L.) 1: a hydroponic screen. TAG. Theor Appl Genet.

[CR55] Proveniers MCG, van Zanten M (2013). High temperature acclimation through PIF4 signaling. Trends Plant Sci.

[CR56] Riboni M, Test A, Galbiati M, Tonelli C, Conti L (2014). Environmental stress and flowering time. Plant Signal Behav.

[CR57] Ritchie ME, Phipson B, Wu D, Hu Y, Law CW, Shi W, Smyth GK (2015). limma powers differential expression analyses for RNA-sequencing and microarray studies. Nucleic Acids Res.

[CR58] Saleh A, Alvarez-Venegas R, Yilmaz M, Le O, Hou G, Sadder M, Al-Abdallat A, Xia Y, Lu G, Ladunga I, Avramova Z (2008). The highly similar *Arabidopsis* homologs of trithorax ATX1 and ATX2 encode proteins with divergent biochemical functions. Plant Cell Online.

[CR59] Seiler C, Harshavardhan VT, Rajesh K, Reddy PS, Strickert M, Rolletschek H, Scholz U, Wobus U, Sreenivasulu N (2011). ABA biosynthesis and degradation contributing to ABA homeostasis during barley seed development under control and terminal drought-stress conditions. J Exp Bot.

[CR60] Seo M, Hanada A, Kuwahara A, Endo A, Okamoto M, Yamauchi Y, North H, Marion-Poll A, Sun T-P, Koshiba T, Kamiya Y, Yamaguchi S, Nambara E (2006). Regulation of hormone metabolism in *Arabidopsis* seeds: phytochrome regulation of abscisic acid metabolism and abscisic acid regulation of gibberellin metabolism. Plant J.

[CR61] Seo DH, Ryu MY, Jammes F, Hwang JH, Turek M, Kang BG, Kwak JM, Kim WT (2012). Roles of four *Arabidopsis* U-Box E3 ubiquitin ligases in negative regulation of abscisic acid-mediated drought stress responses. Plant Physiol.

[CR62] Serraj R, Krishnamurthy L, Kashiwagi J, Kumar J, Chandra S, Crouch JH (2004). Variation in root traits of chickpea (*Cicer arietinum* L.) grown under terminal drought. Field Crops Res.

[CR63] Shan H, Chen S, Jiang J, Chen F, Chen Y, Gu C, Li P, Song A, Zhu X, Gao H, Zhou G, Li T, Yang X (2012). Heterologous expression of the chrysanthemum R2R3-MYB transcription factor *CmMYB2* enhances drought and salinity tolerance, increases hypersensitivity to ABA and delays flowering in *Arabidopsis thaliana*. Mol Biotech.

[CR64] Sharma N, Xin R, Kim DH, Sung S, Lange T, Huq E (2016). No flowering in short day (NFL) is a bHLH transcription factor that promotes flowering specifically under short-day conditions in *Arabidopsis*. Development.

[CR65] Song YH, Shim JS, Kinmonth-Schultz HA, Imaizumi T (2015). Photoperiodic flowering: time measurement mechanisms in leaves. Ann Rev Plant Biol.

[CR66] Tamaki S, Matsuo S, Wong HL, Yokoi S, Shimamoto K (2007). Hd3a protein is a mobile flowering signal in rice. Science.

[CR67] Toledo-Ortiz G (2003). The *Arabidopsis* basic/helix-loop-helix transcription factor family. Plant Cell Online.

[CR68] Tsuji H, Taoka K-I, Shimamoto K (2013). Florigen in rice: complex gene network for florigen transcription, florigen activation complex, and multiple functions. Curr Opin Plant Biol.

[CR69] Uga Y, Sugimoto K, Ogawa S, Rane J, Ishitani M, Hara N, Kitomi Y, Inukai Y, Ono K, Kanno N, Inoue H, Takehisa H, Motoyama R, Nagamura Y, Wu J, Matsumoto T, Takai T, Okuno K, Yano M (2013). Control of root system architecture by DEEPER ROOTING 1 increases rice yield under drought conditions. Nat Genet.

[CR70] Wan X-R, Li L (2006). Regulation of ABA level and water-stress tolerance of *Arabidopsis* by ectopic expression of a peanut 9-cis-epoxycarotenoid dioxygenase gene. Biochem Biophys Res Commun.

[CR71] Wang J, Hu J, Qian Q, Xue H-W (2013). LC2 and OsVIL2 promote rice flowering by photoperoid-induced epigenetic silencing of OsLF. Mol Plant.

[CR72] Wang W-S, Zhu J, Lu Y-T (2014). Overexpression of AtbHLH112 suppresses lateral root emergence in *Arabidopsis*. Funct Plant Biol.

[CR73] Wigge PA, Kim MC, Jaeger KE, Busch W, Schmid M, Lohmann JU, Weigel D (2005). Integration of spatial and temporal information during floral induction in *Arabidopsis*. Science.

[CR74] Wu H, Ye H, Yao R, Zhang T, Xiong L (2015). OsJAZ9 acts as a transcriptional regulator in jasmonate signaling and modulates salt stress tolerance in rice. Plant Sci.

[CR75] Xing D-H, Lai Z-B, Zheng Z-Y, Vinod KM, Fan B-F, Chen Z-X (2008). Stress- and pathogen-induced *Arabidopsis* WRKY48 is a transcriptional activator that represses plant basal defense. Mol Plant.

[CR76] Yoshida S, Fomo DA, Cock JH, Gomez KA (1976). Laboratory manual for physiological studies of rice.

[CR77] Zhang C, Liu J, Zhao T, Gomez A, Li C, Yu C, Li H, Lin J, Yang Y, Liu B, Lin C (2016). A drought-inducible transcription factor delays reproductive timing in rice. Plant Physiol.

[CR78] Zhao X-L, Shi Z-Y, Peng L-T, Shen G-Z, Zhang J-L (2011). An atypical HLH protein OsLF in rice regulates flowering time and interacts with OsPIL13 and OsPIL15. New Biotechnol.

[CR79] Zhou J, Li F, Wang J-L, Ma Y, Chong K, Xu Y-Y (2009). Basic helix-loop-helix transcription factor from wild rice (OrbHLH2) improves tolerance to salt- and osmotic stress in *Arabidopsis*. J Plant Physiol.

