# Supplementary material for: The function of OsbHLH068 is partially redundant with its homolog, AtbHLH112, in the regulation of the salt stress response but has opposite functions to control flowering in Arabidopsis
Source: Plant Mol Biol. 2017 Jun 19;94(4):531–48. doi: 10.1007/s11103-017-0624-6 (PMC5504132; doi:10.1007/s11103-017-0624-6)
Supplement: Supplementary file 1 — Supplementary material 1 (PDF 1762 KB) [file 11103_2017_624_MOESM1_ESM.pdf]

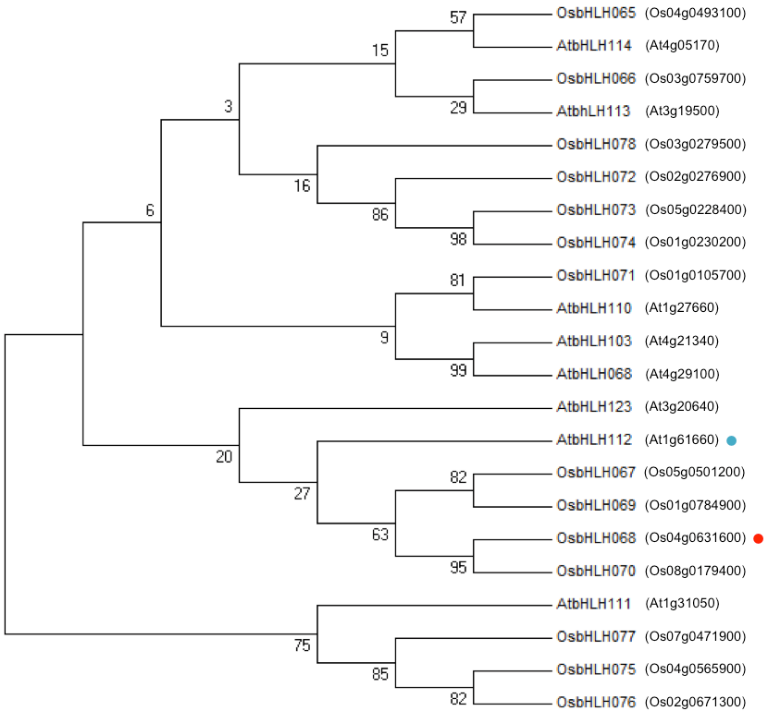

Supplementary Fig. S1 Phylogenetic analysis of bHLH domain sequences among the members of the F subfamily in *Arabidopsis* and rice by the neighbor-joining method. Numbers next to the descendant node indicate the confidence values based on the bootstrap method.

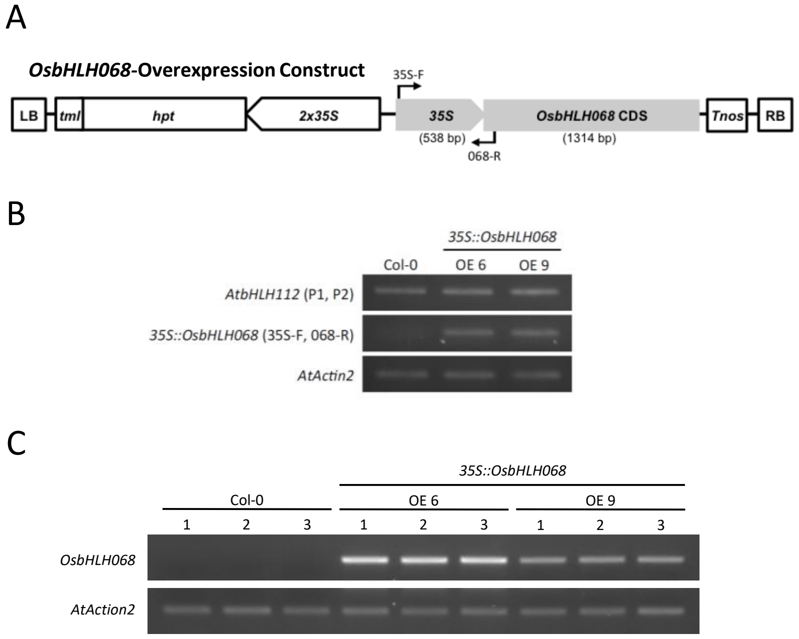

Supplementary Fig. S2 Molecular identification of *OsbHLH068*-overexpressing transgenic *Arabidopsis*. (a) Schematic *OsbHLH068*-overexpression construct. (b) The genetic background of *OsbHLH068*-overexpressing transgenic *Arabidopsis* plants was confirmed by genomic DNA genotyping. The positions of the primers used for genotyping are indicated in **a** and **Supplementary Fig. S3a**. (c) The overexpression of *OsbHLH068* in transgenic *Arabidopsis* plants was confirmed using RT-PCR. The Arabic numerals represent the individual *Arabidopsis* plants.

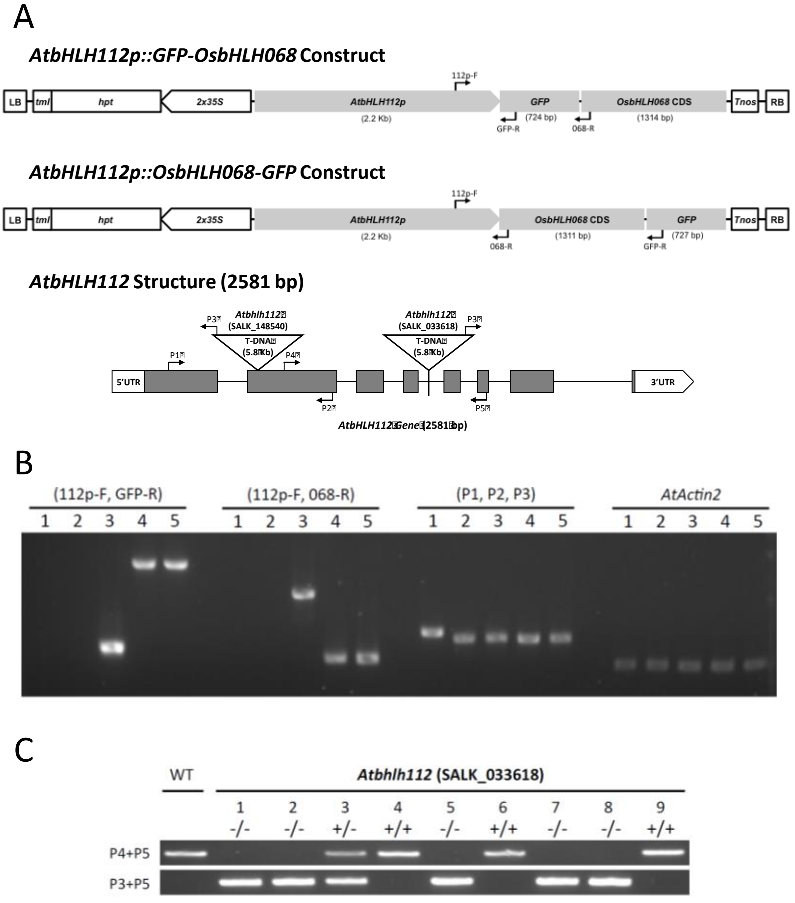

Supplementary Fig. S3 Genotypic identification of homologous *Atbhlh112* mutants and complemented transformants. (a) Schematic of the *AtbHLH112p::GFP-OsbHLH068* and *AtbHLH112p::OsbHLH068-GFP* constructs, and the structure of *AtbHLH112*. (b) The genetic backgrounds of the complemented *AtbHLH112p::GFP-OsbHLH068* and *AtbHLH112p::OsbHLH068-GFP* transformants were confirmed by genomic DNA genotyping. The positions of the primers used for genotyping are indicated in **a**. 1, Col-0; 2, *Atbhlh112* mutant (SALK_148540); 3, *AtbHLH112p::GFP-OsbHLH068* transformant; 4, *AtbHLH112p::OsbHLH068-GFP* transformant (10); 5, *AtbHLH112p::OsbHLH068-GFP* transformant (23). (c) Identification of the homozygous *Atbhlh112* mutant (SALK_033618) by genomic DNA genotyping. The genotyping primer positions are shown in **a**. +/+, non-transformant; +/-, heterozygous mutant; -/-, homozygous mutant.

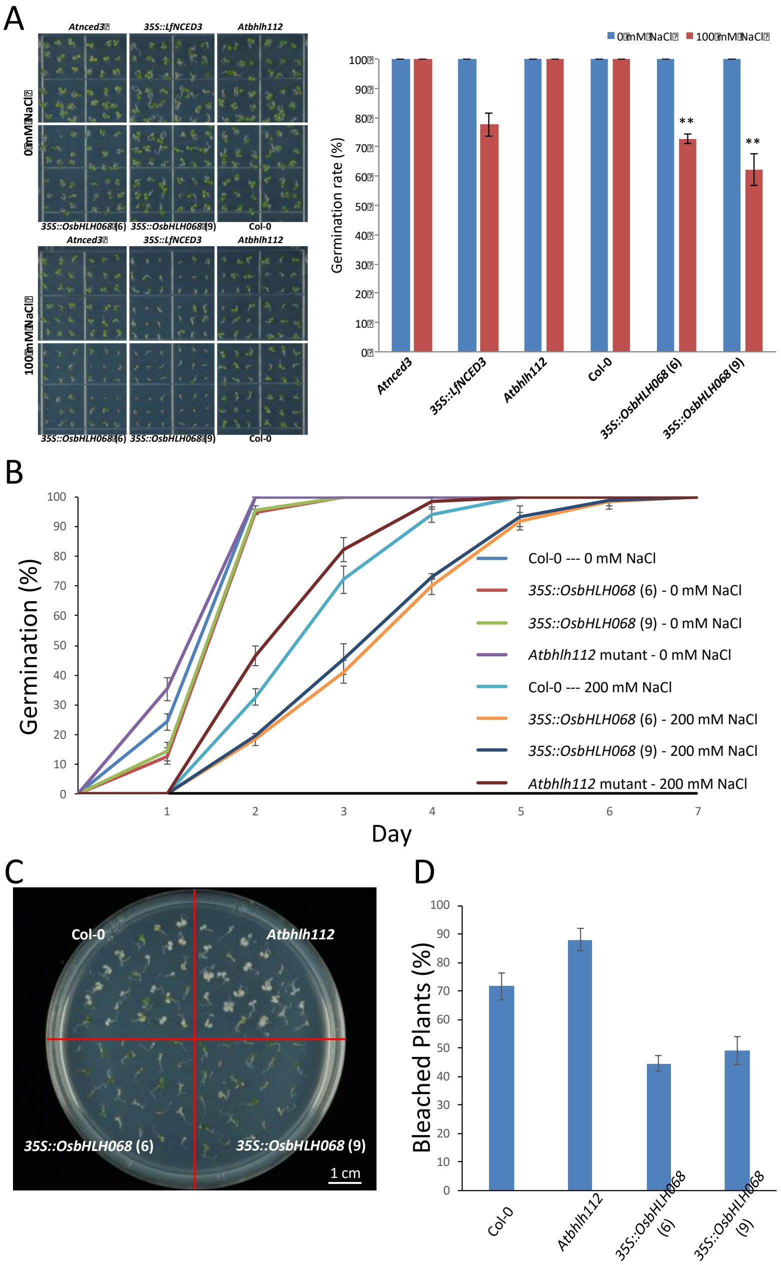

Supplementary Fig. S4 (a) The germination rates of the *Atnced3* mutant, *35S::LfNCED3* transformant, *Atbhlh112* mutant, Col-0, and two *OsbHLH068*-overexpressing transgenic *Arabidopsis* lines under 0 and 100 mM NaCl conditions on day 3. **P* < 0.05; ***P* < 0.01, Student's *t*-test. *Atbhlh112* mutant, SALK_148540. (b) The dynamic changes in seed germination of the *Atbhlh112* mutant, Col-0, and two *OsbHLH068*-overexpressing transgenic *Arabidopsis* lines under 0 and 200 mM NaCl conditions. All germination rates are the mean ± SE of 3 independent biological replicates. Each biological replicate contains 100-125 seeds that were harvested from an individual *Arabidopsis* plant. *Atbhlh112* mutant, SALK_033618. (c) Phenotypic comparison of the *Atbhlh112* mutant, Col-0, and two *OsbHLH068*-overexpressing transgenic *Arabidopsis* lines under 200 mM NaCl conditions on day 14 after seedling. Seedlings were grown on basal agar medium supplemented with 200 mM NaCl. *Atbhlh112* mutant, SALK_033618. (d) Quantification of bleached plants of the *Atbhlh112* mutant, Col-0, and two *OsbHLH068*-overexpressing transgenic *Arabidopsis* lines in c. The values are the mean ± SE of 3 independent biological replicates. Each biological replicate contains 100-125 seedlings. *Atbhlh112* mutant, SALK_033618.

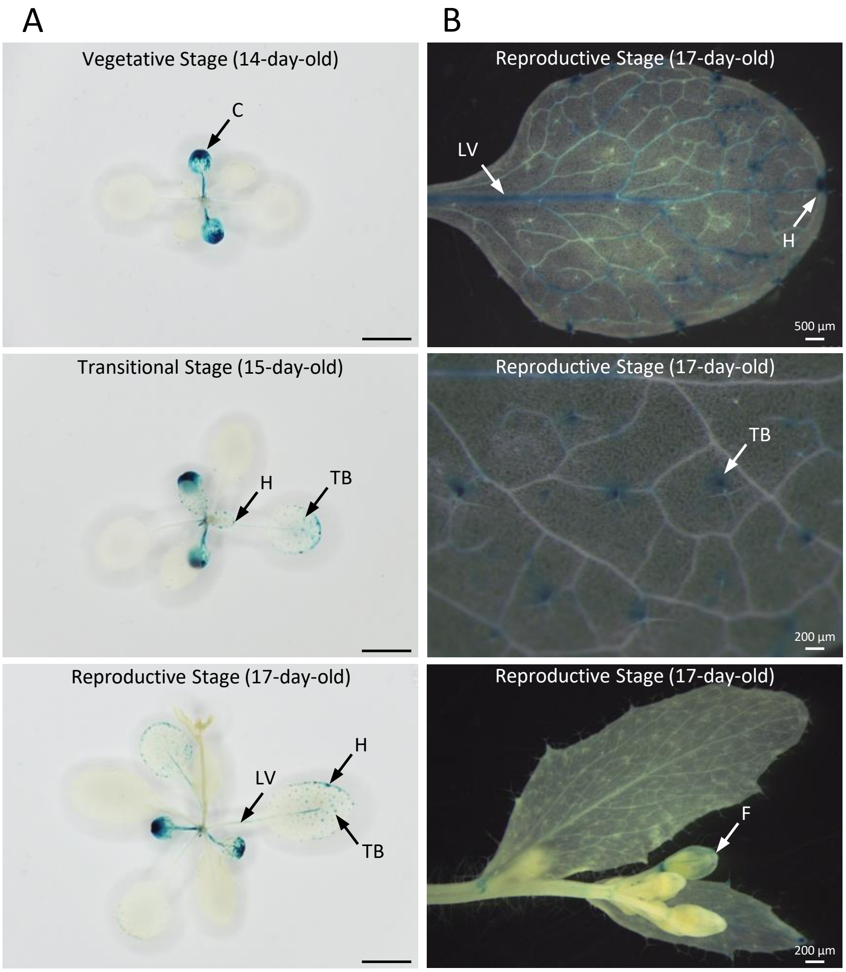

Supplementary Fig. S5 *OsbHLH068* is expressed during both the vegetative and reproductive stages of transgenic *Arabidopsis* plants. (a) Histochemical staining of *OsbHLH068p::GUS* transgenic aerial tissues during the vegetative, transitional, and reproductive stages. Seedlings were grown in soil under LD conditions. Scale bar, 0.5 cm. (b) Microscopic observation of GUS signals in 17-day-old (reproductive stage) transgenic aerial tissues. C, cotyledon; F, floret; H, hydathode; LV, leaf vein; TB, trichome base.

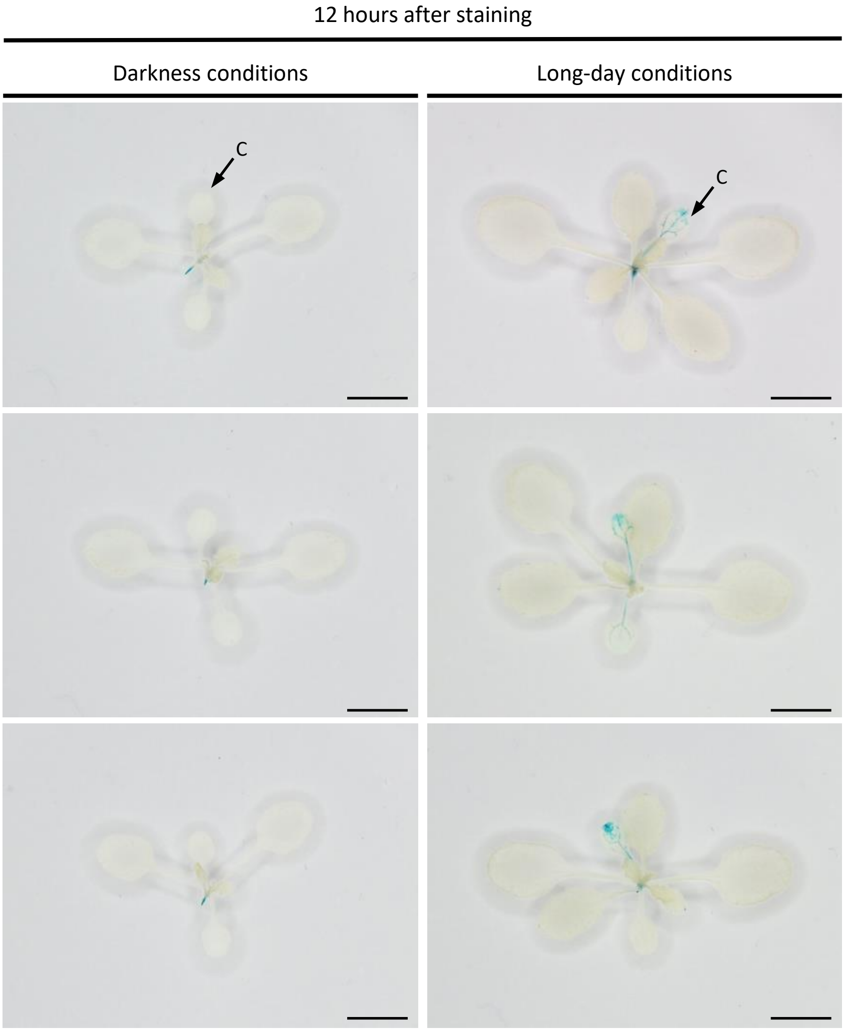

Supplementary Fig. S6 *OsbHLH068* expression is enhanced by light. Histochemical staining of dark- and LD-treated *OsbHLH068p::GUS* transgenic aerial tissues. Seedlings were grown in soil under LD conditions for 11 days and then transferred to darkness or constant LD conditions for an additional 3 days. The samples were fixed 12 h after staining. Scale bar, 0.5 cm. C, cotyledon.

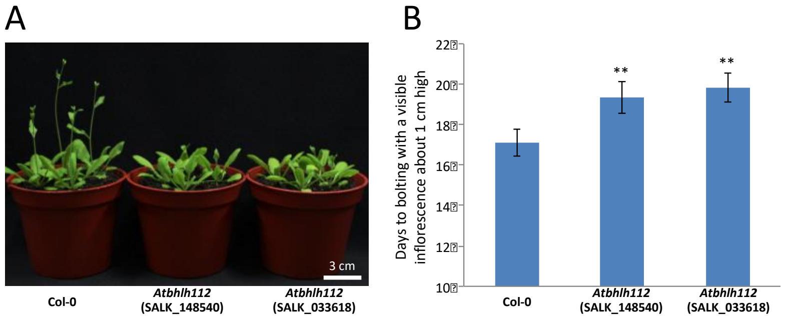

Supplementary Fig. S7 *Atbhlh112* mutants display a late-flowering phenotype. (a) Phenotypic comparison of 19-day-old Col-0 and two independent *Atbhlh112* mutant seedlings. The seedlings were grown in soil under LD conditions. (b) The bolting time of Col-0 and two independent *Atbhlh112* mutant seedlings under LD conditions. The values are the mean ± SE of 12 independent biological replicates. **P* < 0.05; ***P* < 0.01, Student's *t*-test.

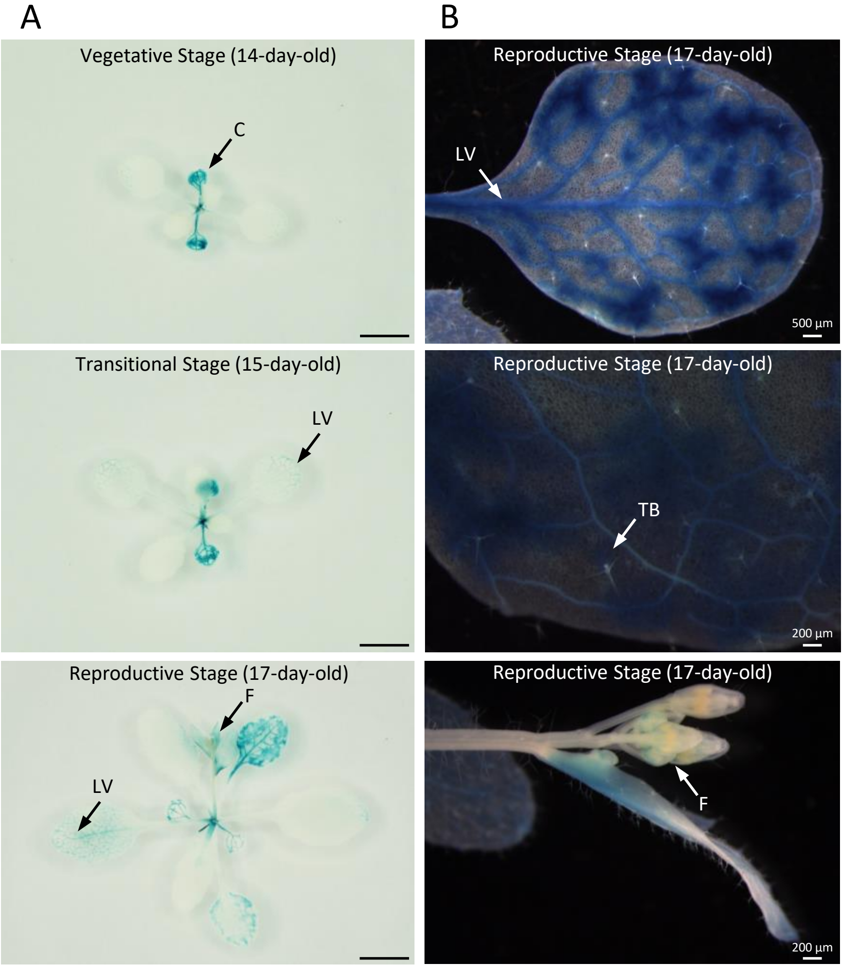

Supplementary Fig. S8 *AtbHLH112* is expressed during both the vegetative and reproductive stag. (a) Histochemical staining of *AtbHLH112p::GUS* transgenic aerial tissues during the vegetative, transitional, and reproductive stages. The seedlings were grown in soil under LD conditions. Scale bar, 0.5 cm. (b) Microscopic observation of GUS signals in 17-day-old (reproductive stage) transgenic aerial tissues. C, cotyledon; F, floret; LV, leaf vein; TB, trichome base.

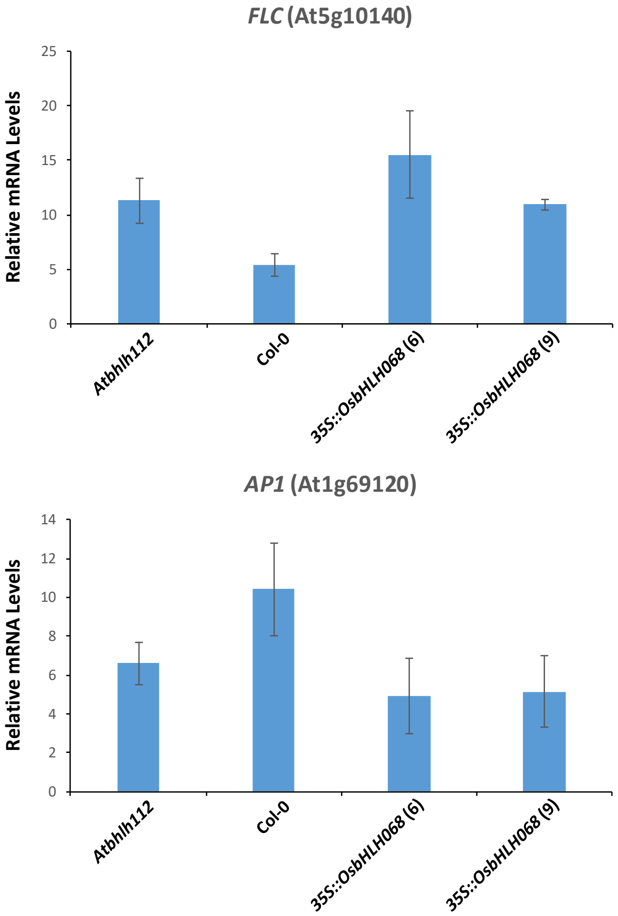

Supplementary Fig. S9 Quantification of *FLC* and *AP1* mRNA levels in *Atbhlh112*, Col-0, and two *OsbHLH068*-overexpressing transgenic *Arabidopsis* lines using qPCR. Total RNA was extracted from the aerial tissues of 17-day-old seedlings. The values are the mean ± SE of 4 biological replicates, each with two technical replicates. *P < 0.05; **P < 0.01, Student's t-test. *Atbhlh112* mutant, SALK_148540.
