# Supplementary material for: The function of OsbHLH068 is partially redundant with its homolog, AtbHLH112, in the regulation of the salt stress response but has opposite functions to control flowering in Arabidopsis
Source: Plant Mol Biol. 2017 Jun 19;94(4):531–48. doi: 10.1007/s11103-017-0624-6 (PMC5504132; doi:10.1007/s11103-017-0624-6)

Biological Process

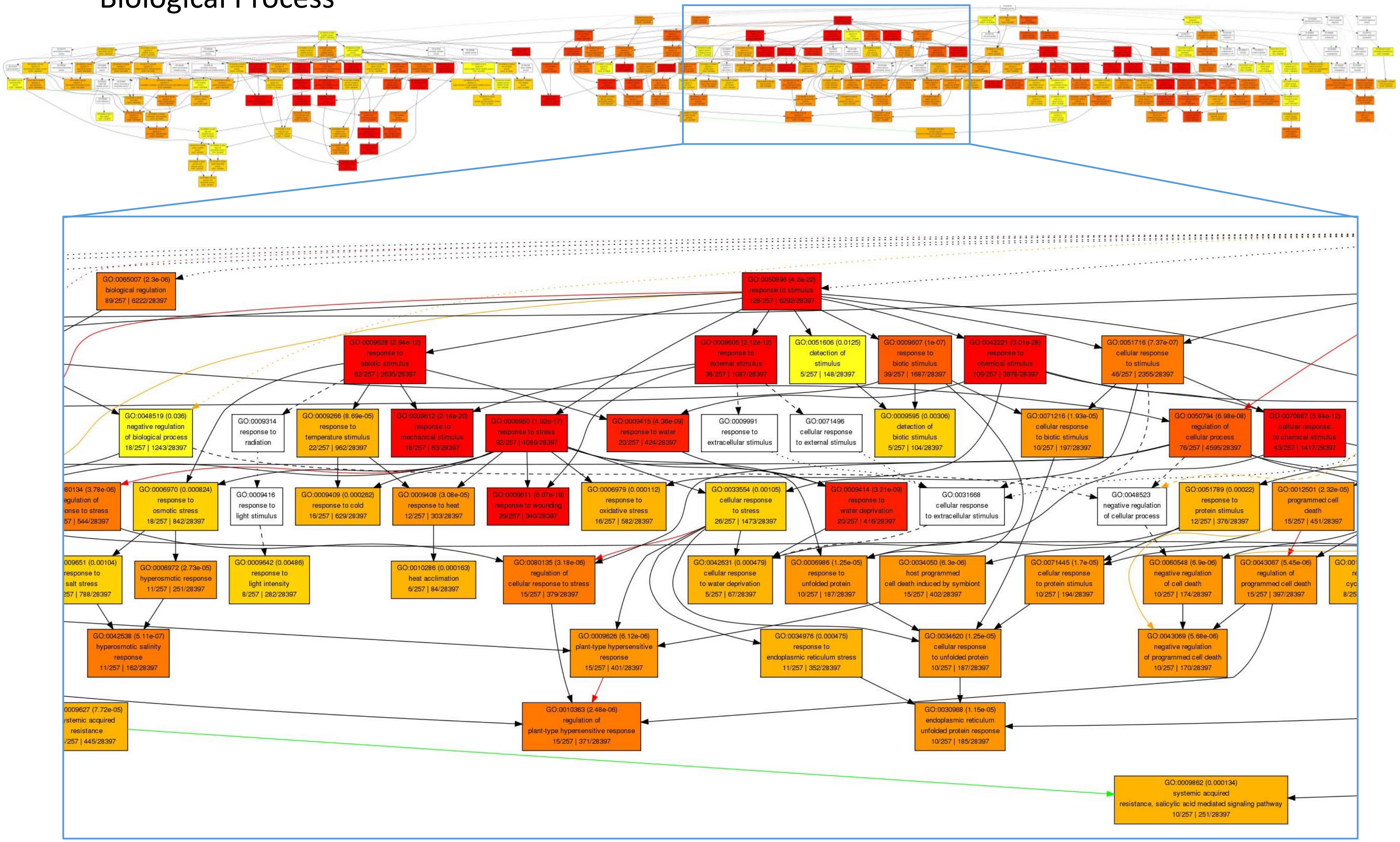

Supplementary Fig. S10 Gene ontology analysis of the common DEGs between *OsbHLH068*- and *AtbHLH112*-overexpressing transgenic *Arabidopsis*.

Cellular Component

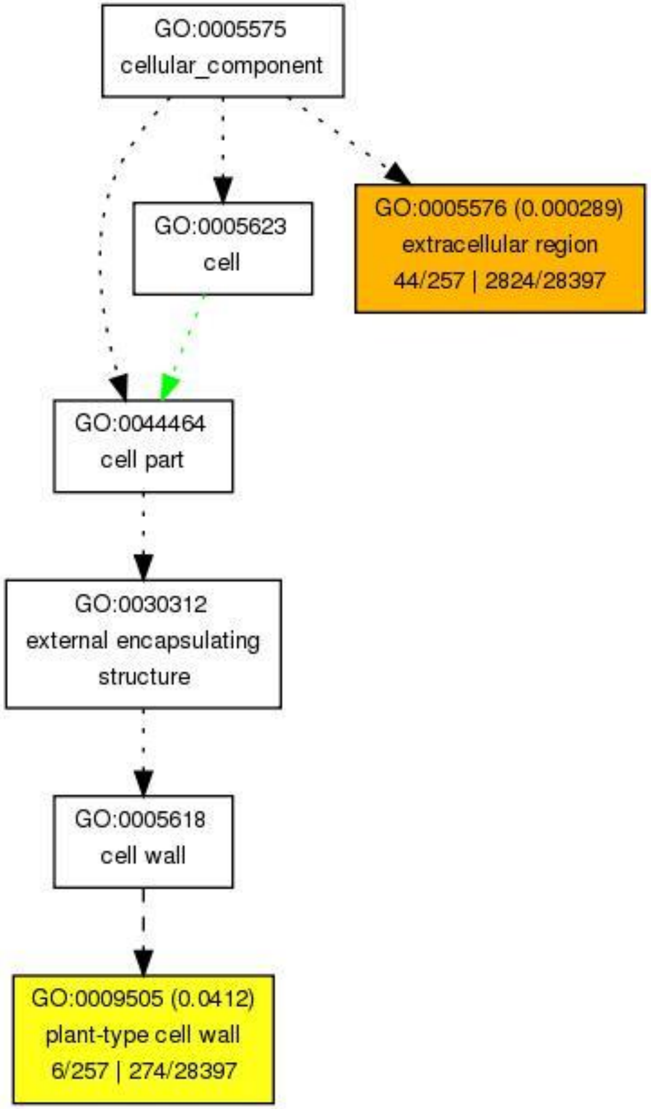

Molecular Function

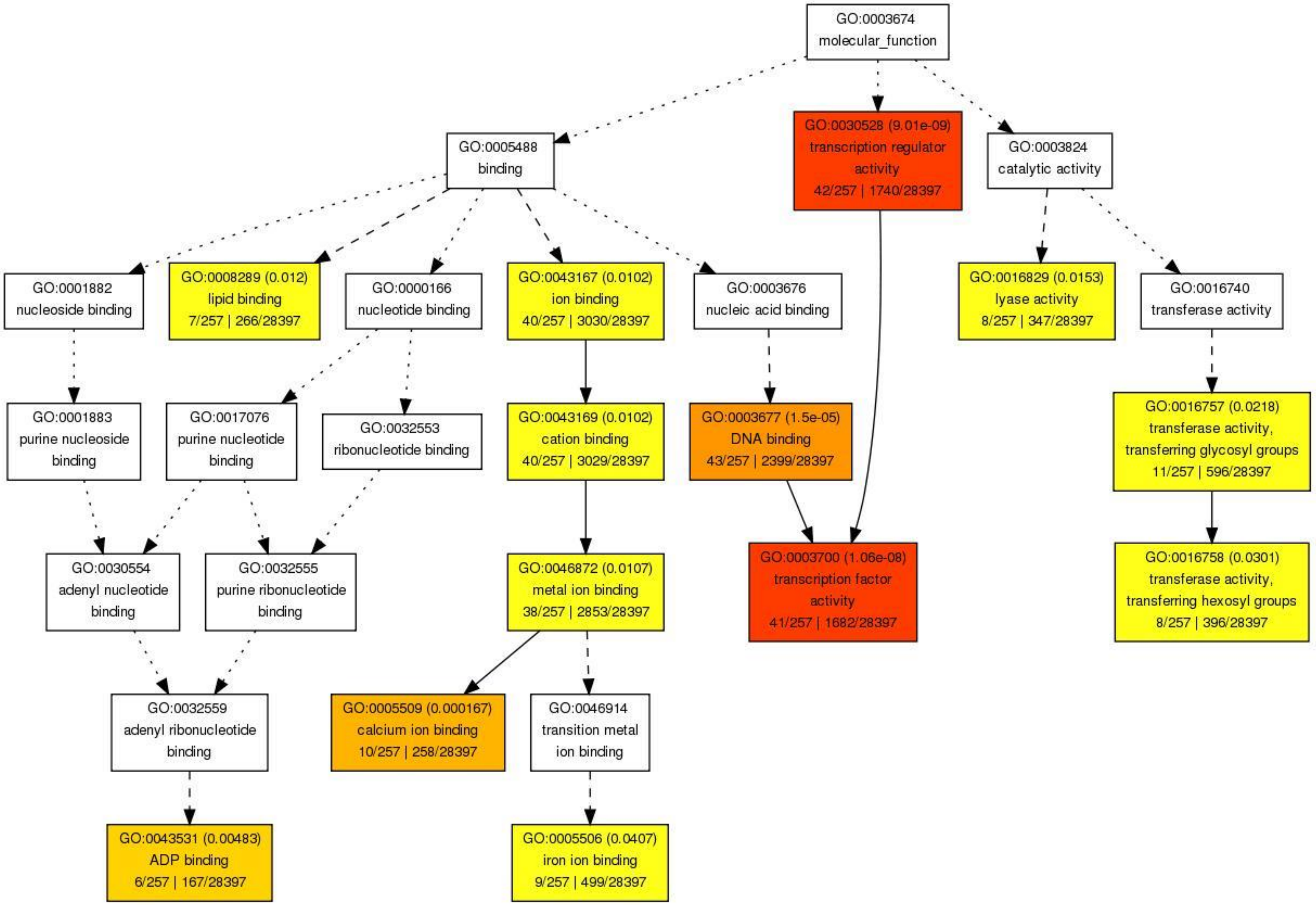

Supplement: Supplementary file 2 — Supplementary material 2 (PDF 815 KB) [file 11103_2017_624_MOESM2_ESM.pdf]
