# Supplementary material for: The function of OsbHLH068 is partially redundant with its homolog, AtbHLH112, in the regulation of the salt stress response but has opposite functions to control flowering in Arabidopsis
Source: Plant Mol Biol. 2017 Jun 19;94(4):531–48. doi: 10.1007/s11103-017-0624-6 (PMC5504132; doi:10.1007/s11103-017-0624-6)
Supplement: Supplementary file 3 — Supplementary material 3 (PDF 173 KB) [file 11103_2017_624_MOESM3_ESM.pdf]

Supplementary Table 1. The 1-kb promoter region (FT, SOC1, Hd3a, and RFT1).

E-box: CANNTG
CAATTG
CAACTG
CAAGTG
CAAATG
CATTTG
CATCTG
CATGTG
CATATG
CACTTG
CACCTG
CACGTG (as known as G-box)
CACATG
CAGTTG
CAGCTG
CAGGTG
CAGATG

>FT (AT1G65480)
ATAATATGGCCGCTTGTTTATAAAAAAAGAAGAGAAATAAAACAATTGATTTGGTTTATATTATTTA
ATTGCAGATATCTTGTACTTAATTCATTTTGAGATAATTTTGCGTATTTGAGTTCGGACATTGGTAG
GTATGGACGATGAAAATAACTGCCTTCATTCTACATGTTTGAGATTTGTTTGTCGACCATATAACAC
AAGCGGCTAGAAAAATAGGTGACTATTCTCAAATGTCCTGGTTCTATCTAACCTGAAGGATCCCTTG
TTCTACCTGGTATAACTACAAGAAAAGGCTGTTTTATAAAATATATCATTTCTTCTATGATTTCTAT
CTCAACTTTTTTTTTAGTTTTTTTCTTTTAGAACGTTTTCGCTTTCGAATTTTTTAAGAATTTTTTT
TTTCTTTATATCTCTAAATAATAGTTTTAAAAACATAACAAGTGATACTGTAATTTTTTTTTCTCTA
TAAACTTGGCGGTACCCTACTTTTTTCTTATTTTTATTAATAATCTTACTTTGTTATGATAAATTAT
GTTAATATATGGCAAAACTAAATATATATGAAACTACTGATTACTCATTATCATAATTAATATCTTT
GTATAAAAGTAAATAATATTTAGTCGAGAGAGGTATCTTGTTAAAGGAAATAAATCAAAAATTAGTG
GCTACCAAGTGGGAGATATAATTTGGAATATTTCCAGTGTATTAGTGTGGTGGGTTTGGAATACCAC
AAACAGAAATAAAAAGAAAGAAAAATATGAAATAAGACGACAATGTGTGATGTACGTAGAATCAGTT
TTAGATTCTAGTACATCAATAGACAAGAAAAAGATTGTGGTTATGATTTCACCGACCCGAGTTAATG
CAAATCCGAAACAGTATAAATATGTGTAGAGGGTTCATGCCTATGATACAAATTAAAGAAGCAGAAA
CAAAAACAAGTAAAACAGAAACAATCAACACAGAGAAACCACCTGTTTGTTCAAGATCAAAG

>SOC1 (AT2G45660)
TGTCCATATGTATCAAAATATGCTATTTTTGGTCTTTCTTAAGGCTTTTTTCCAAAATAAGTAAAGG
ATGAGGTTTCAAGCGTCCATCATATTTGCGACACATATGACTGACTATTTAGCTCCTCCCTCTTTCT
TTCTCTTATTTTATTATCTTTCTCCAAGAAATAAAATAGAAAAGAAAATATATATGGTTTCACAAAC
ACCATTACCATAACTACAACGAGAAGAGGATCTTTTTTAAGGAGAAAAGCAGAGAGAGAAGAGACGA
GTGTGTGAAGTTTTTTTGTCTTTTGTTTCTTTTATTACACACAAATAGATGAAACGAGGAAAGCTAC
TTCTTTTGCTACTTCCATAAAAAGGTTCTTCCTTTCGCAGAGAATCAACTTTGATCATCTTCTTCCT
TCTCTTTCTTTCTTCTTCTCCCTCCAGTAATGCTTATATAGTCTCCTCCTATATCTCTACCTATACA
TACACAAACCCTTTATCCTCGAAAGCTTCCTCCTGGTTAGGTTTTTATCAAACCCTTTTAGCCAATC
GGTAAGATCTCTTCGTCATGATCTTTTCTTTTTTTCTTTTGCTTTGTACTCTGATGGATCTATAAACT
TATATGGGTTTGGTTTCATTTGGTTCGATTTGATGTGTTTGGTTTCTTTGTCCTAAATCTCATGAAA
GGAGGTTGCATCCTTCAATTAAACCGATAACAAAAGTTTCCATTACAGACTTATAGATCAGATACTT
TAGATTGTTTTGCTTTTTGGGTACTTAATCTTTCGTTGACTTCATCAGTCTTCTCCCACCCAAACAA
AAAAGTCATATTTCGATCATATCTTCATTTTTTTAACCTACTCTCTTTGATTCATATATGAAATGGG

TTGTTTTATGTGTGTGACTAATCTTGTTATTGAGGTGGTTGCACCATTGATCTACCGTTTTCTTCAA
TTTTTGAAAAAATAATTTTATTTTTTTTCTGTGTGCAAGGGAAATTAACTAAAGAAGAAGAT

>Hd3a (Os06g0157700)
AGGGAGTACTACATATAACCTCATTTTTTTACGGGTTGATAAGTTGCGGAAAAACCAACAAATTAGC
AACAAATATGAGTAAAACTTGTATACATGTGTCTTCTTAGCGATTTAAAAATCAATGCTGAAAATAA
ATTACAATAAAATTAAAAATCTCAAGATAATCTCTAAAATGTAGTTTTAAAATTTAAATTTTGATTG
CGACTGATAAGAAAAAAAAAACAAATGATGGGAGGCTATATCAACTGTCAAACTGGCTAATTTAGAAA
GACGACATCGAATTCCTACAGATTGGAGGCAGCAAAAGAGAGCCCGTCTCTGCCGGGTGCGTGCATG
ATGCTCGATCATATCCCATCTCTCCTCACTTCATCATCAACAAGAACAAGAGGAACTATATAGCTGC
AAGATCTAGACTGAAACTACTATAGCAACTAACTGTACTGTAGCTAGATTACGCTTAGCTATAGCTG
CTGCTGCATCTATCTGTAAATTCCAACTACGACGTCGACTGCTGCAAGCTAGCTTGGCCGGCTAGCT
AGATAGCTAGCTAGCTCAAAAGAGAAAGCTTTGCGAAATAGGAGTAACTAGCTAGCTCTAGCTAGGG
CGCCATCGATGCGTGACCACCGAGCTCGCCTCGTCCACACGTACAGGAAGACGATGCAGAAAGCGGC
CGGCATGCAGTACACTGACCGAGCTAAGAGAGAGAGAGAGATGATATTATTCTTCTGCAGCTAAATT
AAAGTGAAAGTTGGACATGGACATGGACATAGTAATTTTGCATGGCCATCATCTTGCCCTCCTATAT
AAAGCGGCCATCTCACTCTCAACCACAGCTCGATCCAGCAGCCCTGCACCACACACAGTTCAGCTAG
CAGATCACCTAGCTAGATAGCTGCCTCTATCACAGTATATTTGCTCCCTGCAACTTGCTGCTGCTGC
AATAGCTAGCAGCTGCAGCTAGTAAGCAAAACTATAAACCTTCAGGGTTTTTTGCAAGATCG

>RFT1 (Os06g0157500)
AGAATCAAAAGCTCTCAAAATAGTTCAGCTGTTGGTCCAGATTCTAAAAAGCTATAGTTATAGAATT
TATAAAAGAACTAAAAGCCAGAAGCTAGGTAACCAGCTTTTCCAAACTCTCAGAGCCTGGTTACCAA
ACAGCTGTTCTTAACTCTGCCAAACATGCCCATAGTTAGCTTCATTATTTTACTGCTAGCTTCATTT
ATTTTGGACGGGGTTAGTACATATGTATCTGTCTCGAAATCGCCTCTGTAAATGGGGTCAGTTTCAG
CACTTCTATCGACATGAAGAAGATAGAAAGGGCCATGCGAGAGAGAGTGTGTGAGAAAGAGAAGATT
CTACCGGGCACAACAAAGTGTGTAAATGCATAAAGAAAAGATTCTACTGGGCACATCATAGTAAGGC
TATTCTCAGCTCAATAACTAGGATGGTGTCCATAGCATTAAATAAGCTGCTACCTAATATGAAAAAT
GATGTGGCAAGTGAGTAAATGAGGAAAGAGAAGAAAACAAGGTCTTGCATGAGACATGGTTTCTACA
CAACATTGAAGACATCATGTGAGATAAATTTAATTTAAGCATGAAAAAGTGGTGTTTGTATTGAAAA
AGTAGTGTCTAATACTAGTTTATTGATGATGTGAAGTTTATGAAAACTACCGTGTAGTGTCTTGGGT
TGGTACTGCCTAAAGGGGCCGGCTAGCTATATAAACTGTACAGATGCAGAGAAGCTTGCACAACTAG
TGCATCCCTGTCACTGTTTGGCTAGCTTAACCTTCCTGACATCTATCCTCTGGATTGAACGGCAGGA
GATACCTAAGCTAGCTAGCAATCTCTATCGATCTGTTTGTTTACATGTTCAGTTAAAGGTTACTGAG
AAATGCCTAGAGTTTTTCCGGCTAGCTTCATAAGTTAGTGGGTTAGCTGACCTAGATTCAAAGTCTA
ATCCTTTTATTTATTTGATATTAGATATCCTAACGTTTTTAGTTAGAGGTTATTAATTTGAC

Supplementary Table 2. Primer sequences.

Genotyping	Primer Name	Sequence (5'-3')
	35S-F	AGAGGACCTAACAGAACTCG
	068-R	CACGCCATGAGAGCTCGTCGTAC
	P1	TTCGGATCATTTCTTGTCACC
	P2	GTAGATATTATCTGCGGAGTGG
	P3	ATTTTGCCGATTTCGGAAC
	P4	GAACCTTTTACGATCCCGAACC
	P5	CAGTGACTTGGTCGTGAAGG
	112p-F	CACTTCTTCGAAAAGCTAAGCAGC
	GFP-R	GTGCAGATGAACTTCAGGGTCAGC

RT-PCR	Primer Name	Sequence (5'-3')
OsbHLH068 (Os04g0631600)	OsbHLH068-RT-F	GTACGACGAGCTCTCATGGCGTG
	OsbHLH068-RT-R	GGCTCCATGAGGCTCCTCAGCAG
OsActin1 (Os03g0718100)	OsActin1-RT-F	GGAACTGGTATGGTCAAGGCTGG
	OsActin1-RT-R	CACACGGAGCTCGTTGTAGAAGG

q-PCR	Primer Name	Sequence (5'-3')
OsSOS1 (Os12g0641100)	OsSOS1-Q-F	GACACTGGGAATGTTCTATGC
	OsSOS1-Q-R	CCATGAAGCACCGTGCCTCTCG
OsbHLH068 (Os04g0631600)	OsbHLH068-Q-F	TGCTGGTCTGCATGAGTTTC
	OsbHLH068-Q-R	GCTTGCTGCAGTGCTAGGTT
OsActin1 (Os03g0718100)	OsActin1-Q-F	CTCAGCACATTCCAGCAGATGTG
	OsActin1-Q-R	GATAACAGCTCCTCTTGGCTTAGC
MYB4 (At4g38620)	MYB4-Q-F	CAAGGGCATGGAAAGTCAAC
	MYB4-Q-R	TTGCTGCTACCTCCGACTAC
PP2C5 (At2g40180)	PP2C5-Q-F	ATCTGATGGCCTGTGGGACA
	PP2C5-Q-R	CCGATAGCTCAGCCAGTTTC
CBF2 (At4g25470)	CBF2-Q-F	GCTCCGATTACGAGTCTCCG
	CBF2-Q-R	TCTTCCTTCCCGCTGGTTTC
ASC11 (At4g08040)	ASC11-Q-F	CCAGGCTCATCGTGTCATTGCGA
	ASC11-Q-R	GCAACCTCCATCGTTTGGTCCGA
LFY (At5g61850)	LFY-Q-F	CGGCTTAGATTATCTGTTCCACTTG
	LFY-Q-R	ACTCGCTCCTGATTTCTTCGC
SOC1 (At2g45660)	SOC1-Q-F	CTCTTGGGAGAAGGCATAGGAA
	SOC1-Q-R	TCAAGCTGTTGCTCAATCTGTTG
FT (At1g65480)	FT-Q-F	CAACCCTCACCTCCGAGAATAT
	FT-Q-R	TTGCCAAAGGTTGTTCCAGTT
AtActin2 (At3g18780)	AtActin2-Q-F	GTGGATTCCAGCAGCTTCCAT
	AtActin2-Q-R	GCTGAGAGATTCAGATGCCCA
